# Supplementary material for: Quiescent horizontal basal stem cells act as a niche for olfactory neurogenesis in a mouse 3D organoid model
Source: Cell Rep Methods. 2025 May 28;5(6):101055. doi: 10.1016/j.crmeth.2025.101055 (PMC12272253; doi:10.1016/j.crmeth.2025.101055)
Supplement: Document S1. Figures S1–S5 [file mmc1.pdf]

Cell Reports Methods, Volume *5*

Supplemental information

Quiescent horizontal basal stem cells act as a niche for olfactory neurogenesis in a mouse 3D organoid model

Juliana Gutschow Gameiro, Constantin A. Hintschich, Agnès Dekeyser, Valérie Hox, James E. Schwob, Eric H. Holbrook, Marco Aurélio Fornazieri, and Brian Lin

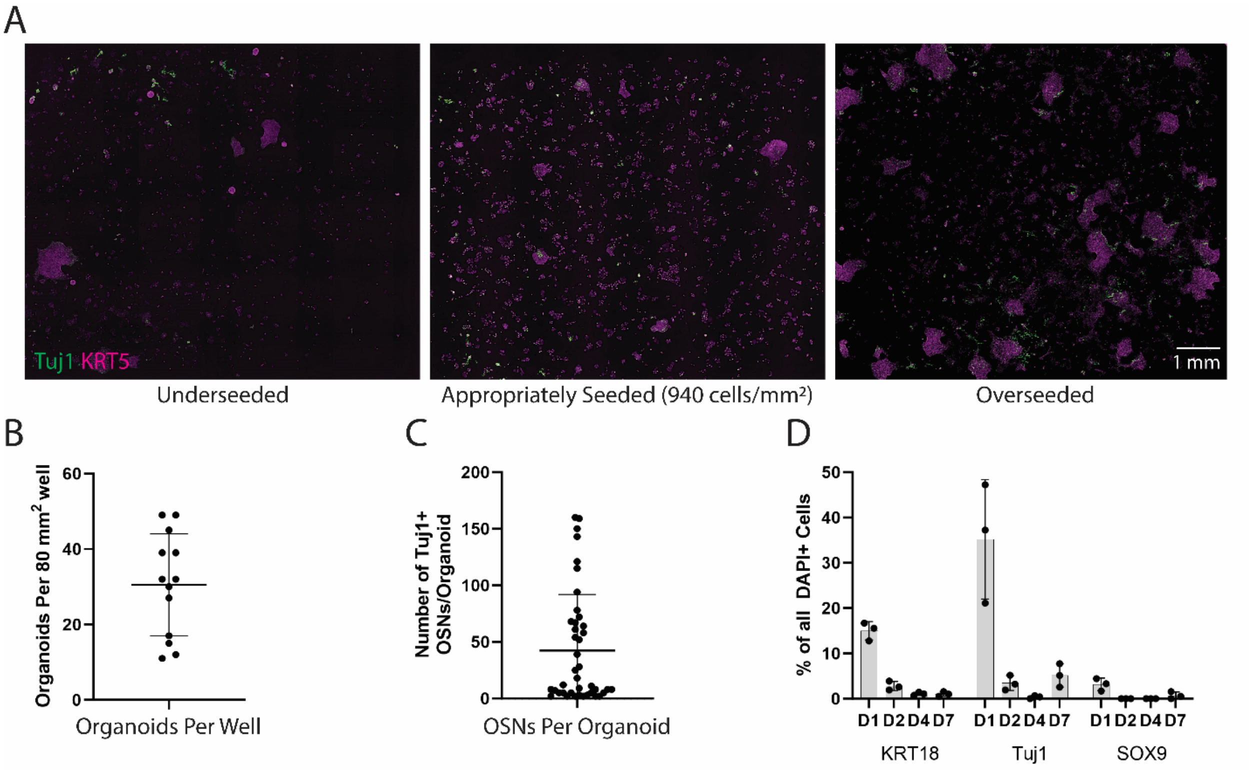

Figure S1: Optimization of organoid seeding and characteristics, related to Figure 1. (A) Representative images of organoid cultures that are underseeded, appropriately seeded, and overseeded. Images are of the entire well of an 8-well chamber slide. Underseeded conditions are uneven and have low numbers of organoids, while overseeded conditions have organoids that merge together, compromising the ability to quantify individual organoids. (B) Quantification of the number of organoids found per well of an 8-well chamber slide when seeded at 940 cells/ mm^2 across 4 distinct biological replicates. Each dot represents a single chamber well. (C) Quantification of the number of Tuj1+ nuclei found per organoid across four distinct biological replicates. Each dot represents a single organoid that was counted. (D) Quantification of cells that were KRT18+, Tuj1+, or SOX9+ in a timecourse starting the day after plating, shown as the percentage of all DAPI+ cells. Each dot represents a single chamber well, where random fields, which in total covered 25% of the well, were manually counted.

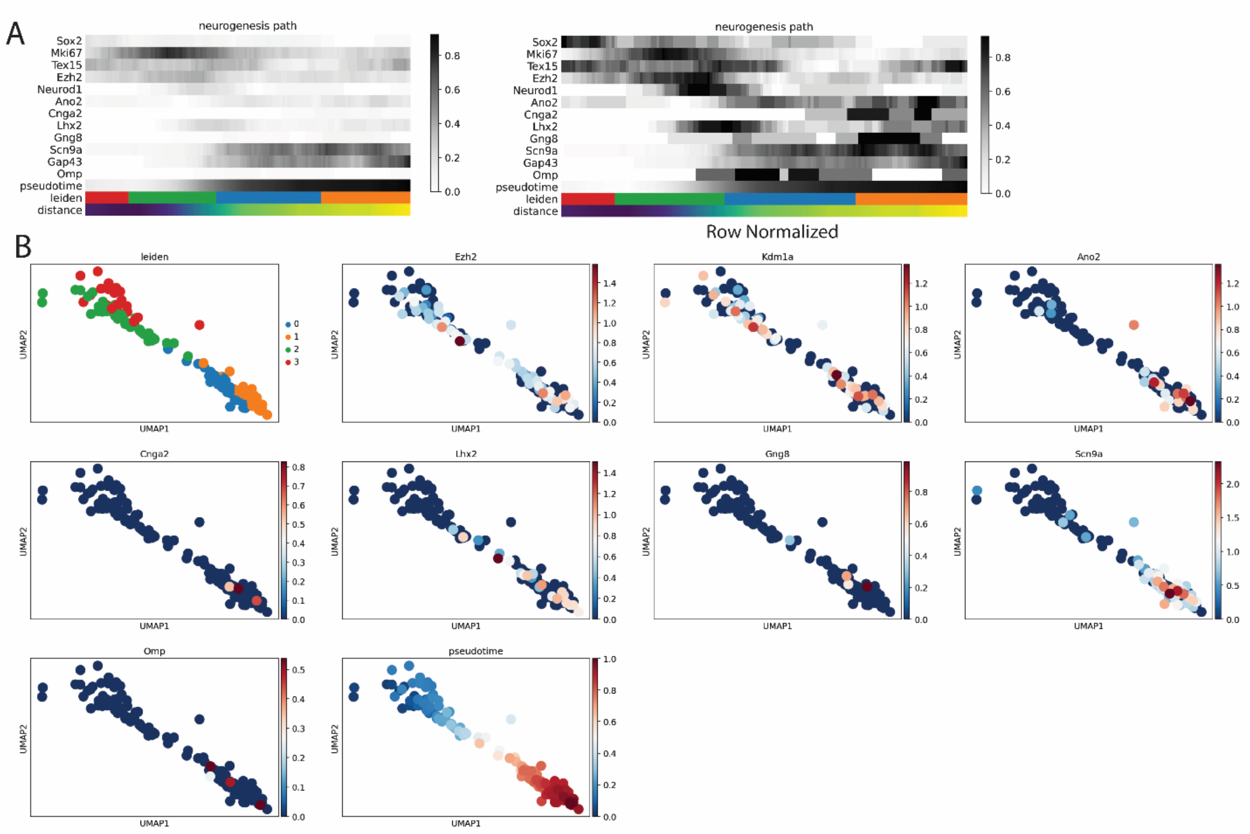

Figure S2: Single-cell RNAseq analysis, related to Figure 1. (A) Pseudotime-aligned gene expression plots of key neurogenic genes. Pseudotime was determined using PAGA, with the Sox2+ cluster set as the root population. On the left, log-transformed gene expression is plotted. On the right, data have been standardized to 0-1 expression to facilitate visualization of lowly expressed genes, which can also highlight noise, as can be seen in the OMP expression. (B) Gene expression of key genes found in neurogenesis and OSN function plotted on a log scale.

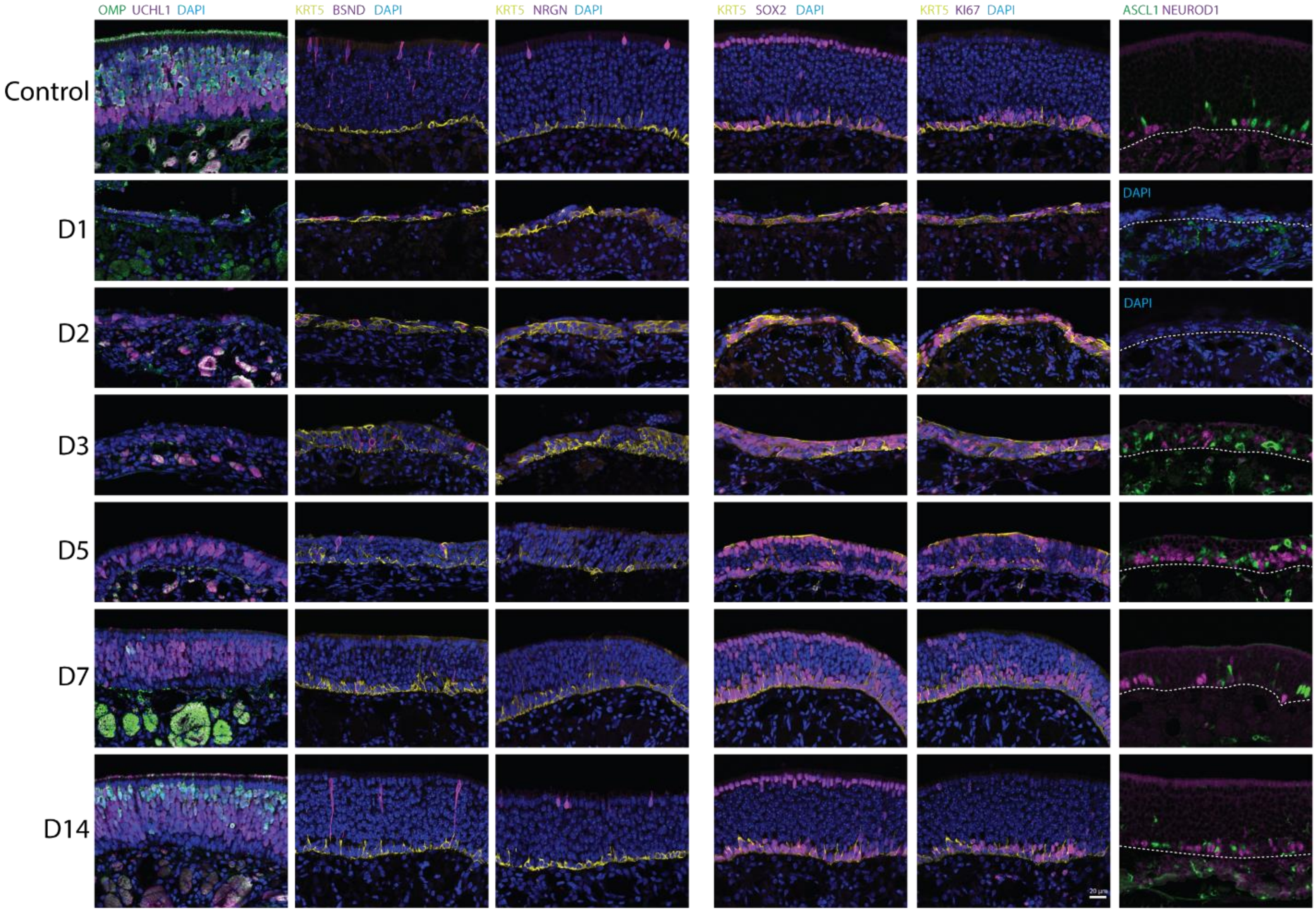

Figure S3: Characterization of methimazole-induced injury, related to Figure 1. Representative immunofluorescent staining for primary cell types found in the OE in a time course of tissue regeneration after methimazole injury. OMP marks mature OSNs, UCHL1/PGP9.5 marks mature and immature OSNs, KRT5 marks HBCs, BSND marks ionocytes/IP3R3+ microvillar cells, NRGN marks TRPM5+ microvillar/tuft cells, luminal SOX2 marks Sus cells, basal SOX2+ cells that are KRT5- are GBCs, KI67 marks dividing cells, ASCL1 marks neuronally committed upstream GBCs and NEUROD1 marks neuronally committed progenitor GBCs. In the right-most panels, DAPI is omitted except for D1 and D2 post-injury for clarity due to color overlap.

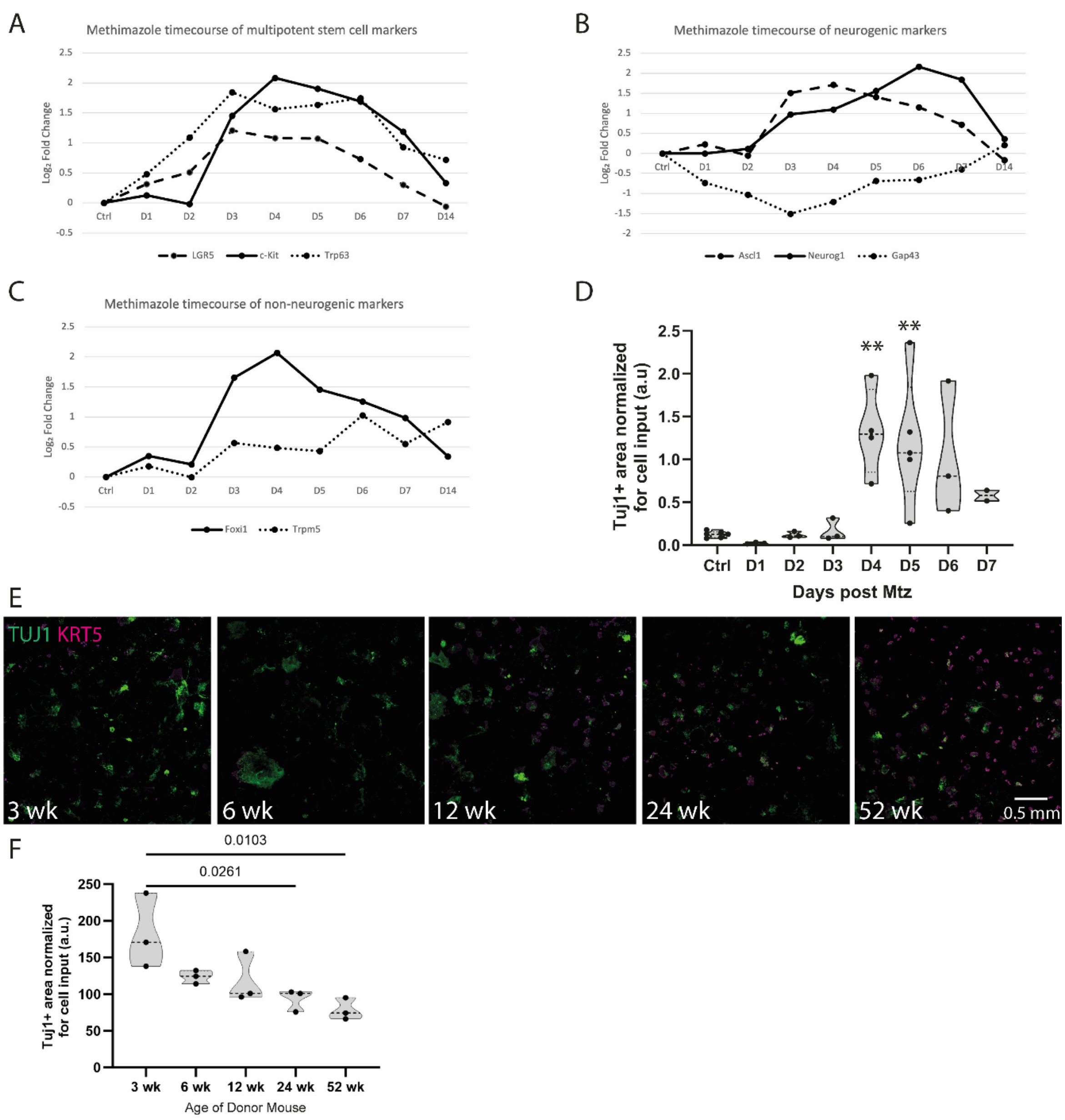

Figure S4: **Optimization of the organoid model, related to Figure 1.** q-RT PCR of OE tissue samples collected at time points post methimazole injury, profiling the gene expression of (A) multipotent stem cell markers, (B) neurogenic markers, and (C) non-neurogenic markers relative to control. (D) Quantification of the efficiency of neurogenesis in the organoid system using tissue harvested at different methimazole injury time points, measured as Tuj1+ area normalized for cell input (a.u.). ANOVA comparison $p = 0.001$, **$p < 0.05$ compared to uninjured control). (E) Representative, low magnification images of organoid cultures stained for Tuj1 and KRT5 isolated from mice aged 3-52 weeks. (F) Quantification of the Tuj1+ area generated by cells isolated from mice of different ages, normalized for cell input (a.u.). ANOVA comparison $p = 0.013$, followed by Dunnett's multiple comparisons test. qRT-PCR n=1 mouse each timepoint, technical triplicates. Post-Mtz experiments were replicated a minimum of 3 times on distinct mice, with each dot representing a different mouse. Age experiments were replicated three times, with each dot representing a different mouse.

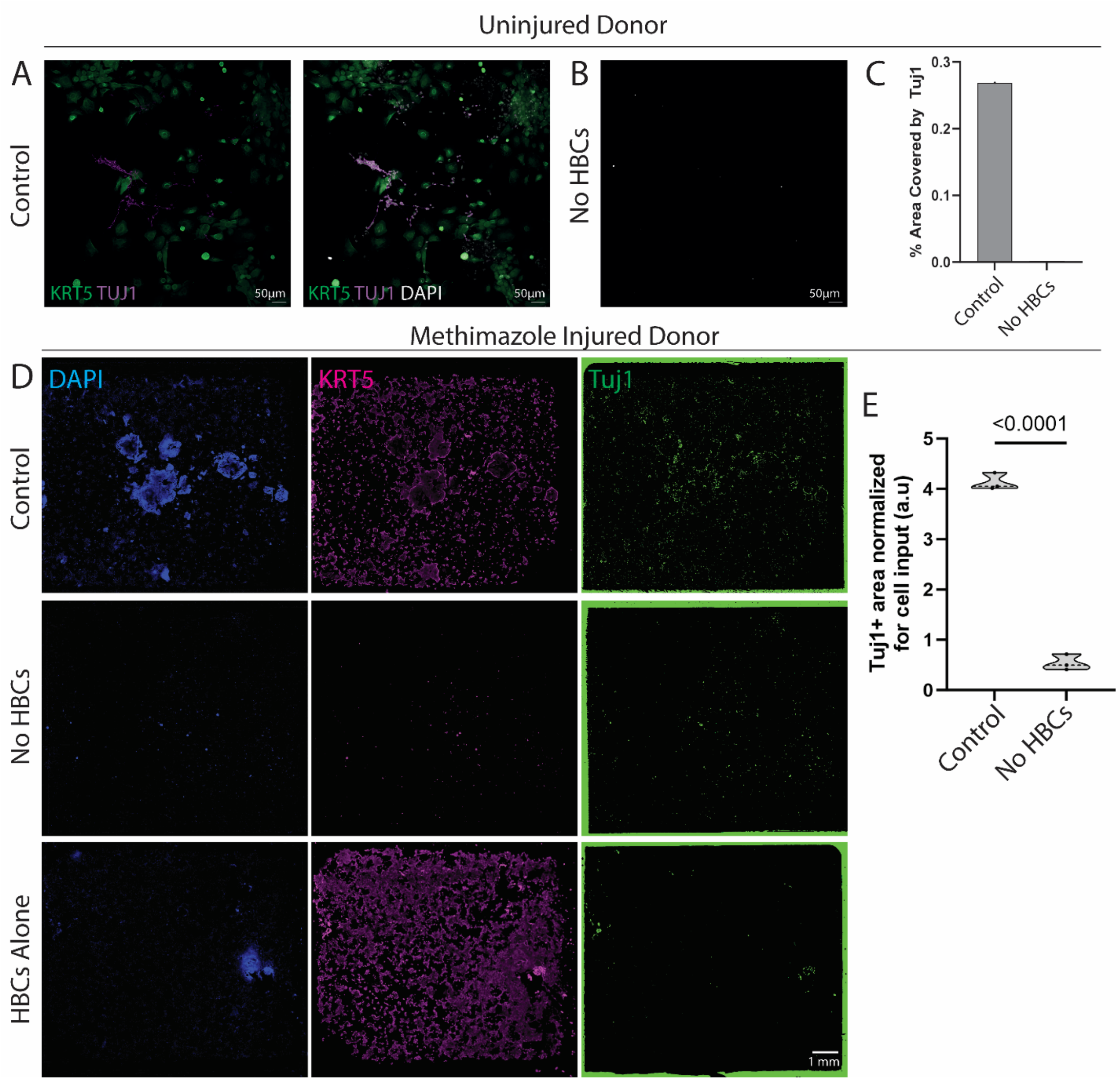

Figure S5: Effect of HBC depletion in chamber slides, related to Figure 3. (A) Representative immunostaining of the positive control (reconstituted HBCs and negative population that all went through the FACS process), and (B) immunostaining of HBC-depleted cultures grown in culture wells. (C) Quantification of the % area covered by Tuj1 in the chamber wells. (D) Representative whole chamber well images of cultures grown from donor animals 4 days after methimazole injury. Seeded cells were unstained controls that went through the entire FACS procedure, samples depleted for HBCs or sorted HBCs alone. Chambers were fixed and stained after 7 days of culture. The green border represents the auto fluorescent markings on the chamber slide itself. (E) Quantification of the Tuj1+ area normalized for chamber well area and cell input. Unpaired t-test, n=3 biological replicates.
