# Supplementary material for: Quiescent horizontal basal stem cells act as a niche for olfactory neurogenesis in a mouse 3D organoid model
Source: Cell Rep Methods. 2025 May 28;5(6):101055. doi: 10.1016/j.crmeth.2025.101055 (PMC12272253; doi:10.1016/j.crmeth.2025.101055)
Supplement: Document S2. Article plus supplemental information [file mmc3.pdf]

Cell Reports Methods

Report

Quiescent horizontal basal stem cells act as a niche for olfactory neurogenesis in a mouse 3D organoid model

Graphical abstract

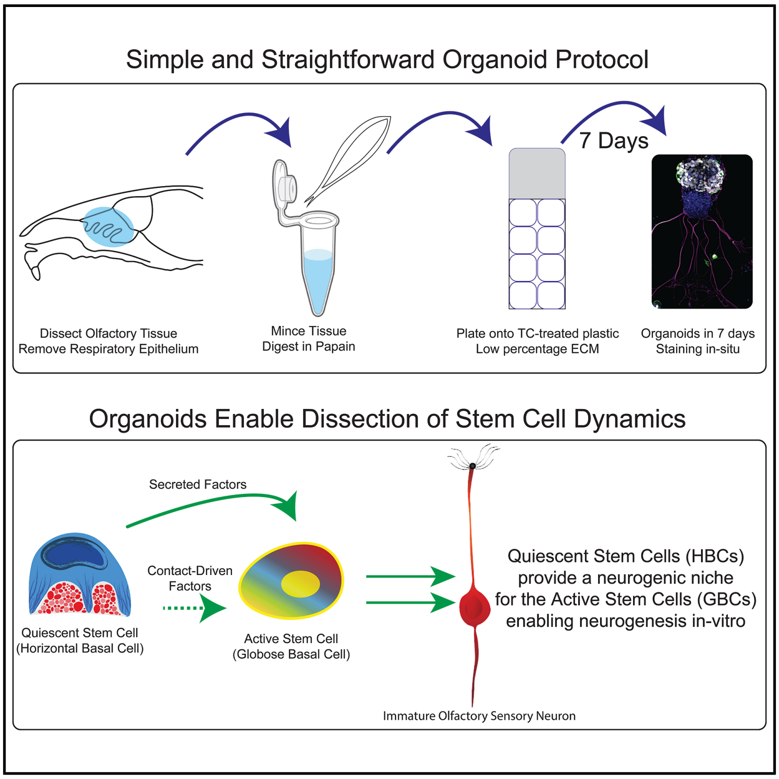

Highlights

- We present an easy-to-use 3D organoid model of mouse olfactory epithelium
- Quiescent stem cells in the tissue (HBCs) do not contribute neurons to the organoid
- Quiescent stem cells act as a neurogenic niche for the active stem cells

Authors

Juliana Gutschow Gameiro, Constantin A. Hintschich, Agnès Dekeyser, ..., Eric H. Holbrook, Marco Aurélio Fornazieri, Brian Lin

Correspondence

marcofornazieri@uel.br (M.A.F.), brian.lin@tufts.edu (B.L.)

In brief

Gameiro et al. describe an easy-to-use 3D organoid model of the mouse olfactory epithelium that supports neurogenesis to an immature olfactory sensory neuron stage and use it to demonstrate that a quiescent stem cell population maintains a neurogenic niche for the active stem cells.

Gameiro et al., 2025, Cell Reports Methods *5*, 101055
June 16, 2025 © 2025 The Author(s). Published by Elsevier Inc.
https://doi.org/10.1016/j.crmeth.2025.101055

CellPress

Cell Reports Methods

CellPress
OPEN ACCESS

Report

Quiescent horizontal basal stem cells act as a niche for olfactory neurogenesis in a mouse 3D organoid model

Juliana Gutschow Gameiro,[1,2,3] Constantin A. Hintschich,[3,4,5,6] Agnès Dekeyser,[3,7] Valérie Hox,[7,8] James E. Schwob,[3] Eric H. Holbrook,[3,4] Marco Aurélio Fornazieri,[1,9,*] and Brian Lin[3,10,*]
[1]Department of Clinical Surgery, State University of Londrina, Paraná, Londrina, Brazil
[2]Health Sciences Graduate Program, State University of Londrina, Paraná, Londrina, Brazil
[3]Department of Developmental, Molecular and Chemical Biology, Tufts University Graduate School of Biomedical Sciences, Boston, MA 02111, USA
[4]Department of Otolaryngology – Head & Neck Surgery, Massachusetts Eye and Ear, Boston, MA, USA
[5]Department of Otorhinolaryngology, Regensburg University Hospital, Regensburg, Germany
[6]Department of Ophthalmology, Ludwig Maximilians University Munich, Munich, Germany
[7]Laboratory of Pneumology, ENT (Airways), and Dermatology (Skin) (LUNS), Institute of Experimental and Clinical Research (IREC), UC Louvain, 1200 Brussels, Belgium
[8]Department of Otorhinolaryngology, Head and Neck Surgery, Cliniques Universitaires Saint-Luc, 1200 Brussels, Belgium
[9]Department of Medicine, Pontifical Catholic University of Paraná, Londrina, Brazil
[10]Lead contact
*Correspondence: marcofornazieri@uel.br (M.A.F.), brian.lin@tufts.edu (B.L.)
https://doi.org/10.1016/j.crmeth.2025.101055

MOTIVATION The olfactory epithelium is one of the few tissues in the mammalian body that facilitates a life-long capacity for neuronal regeneration, but until recently, it has been generally underappreciated. We had two guiding motivations for the work presented here. First, we strove to describe an easy-to-implement and -replicate methodology that did not require unique animal models or fluorescence-activated cell sorting to lower the bar for other researchers to adopt our model for various studies. Second, we wanted to move toward viable *in vitro* screening methodologies, ultimately enabling the development of treatments for olfactory dysfunction.

SUMMARY

The olfactory epithelium contains two basal stem cell populations that facilitate the usually life-long ability for neuronal regeneration that is required for maintaining our sense of smell. Horizontal basal cells (HBCs) are generally quiescent and only become active after direct injury to the epithelium that eliminates more than just the olfactory sensory neurons (OSNs). Globose basal cells (GBCs) lie apical to HBCs and are solely responsible for the generation of olfactory neurons in the undamaged epithelium. Understanding how these two neurogenic stem cell populations are regulated as OSNs are replenished is hampered by a lack of robust culture models. Here, we report the development of a 3D mouse organoid model that recapitulates the neurogenic cascade, forming immature OSNs while maintaining both HBCs and GBCs in culture. We use this model to demonstrate that, despite their relative quiescence, HBCs form a critical niche for the emergence and composition of the organoid.

INTRODUCTION

The mammalian olfactory epithelium (OE) enables our sense of smell through a population of olfactory sensory neurons (OSNs) found in the epithelium, which is usually abundant and composed of hundreds of odorant receptor-defined types. OSNs are in direct contact with the outside environment, rendering them vulnerable to injury and exhibiting variable lifespans.[1–5] Accordingly, olfactory neurogenesis is a lifelong process capable of maintaining the full complement of OSNs and regenerating a normal or near-normal population of neurons after injury. Thus, sensory function can be maintained and even restored, which contrasts with the very limited ability of the central nervous system (CNS) for regeneration. Underlying the capacity of the OE for robust neurogenesis is the lifetime persistence of two multipotent stem cell populations within the OE, the horizontal basal cells (HBCs) and the globose

© 2025 The Author(s). Published by Elsevier Inc.
This is an open access article under the CC BY license (http://creativecommons.org/licenses/by/4.0/).

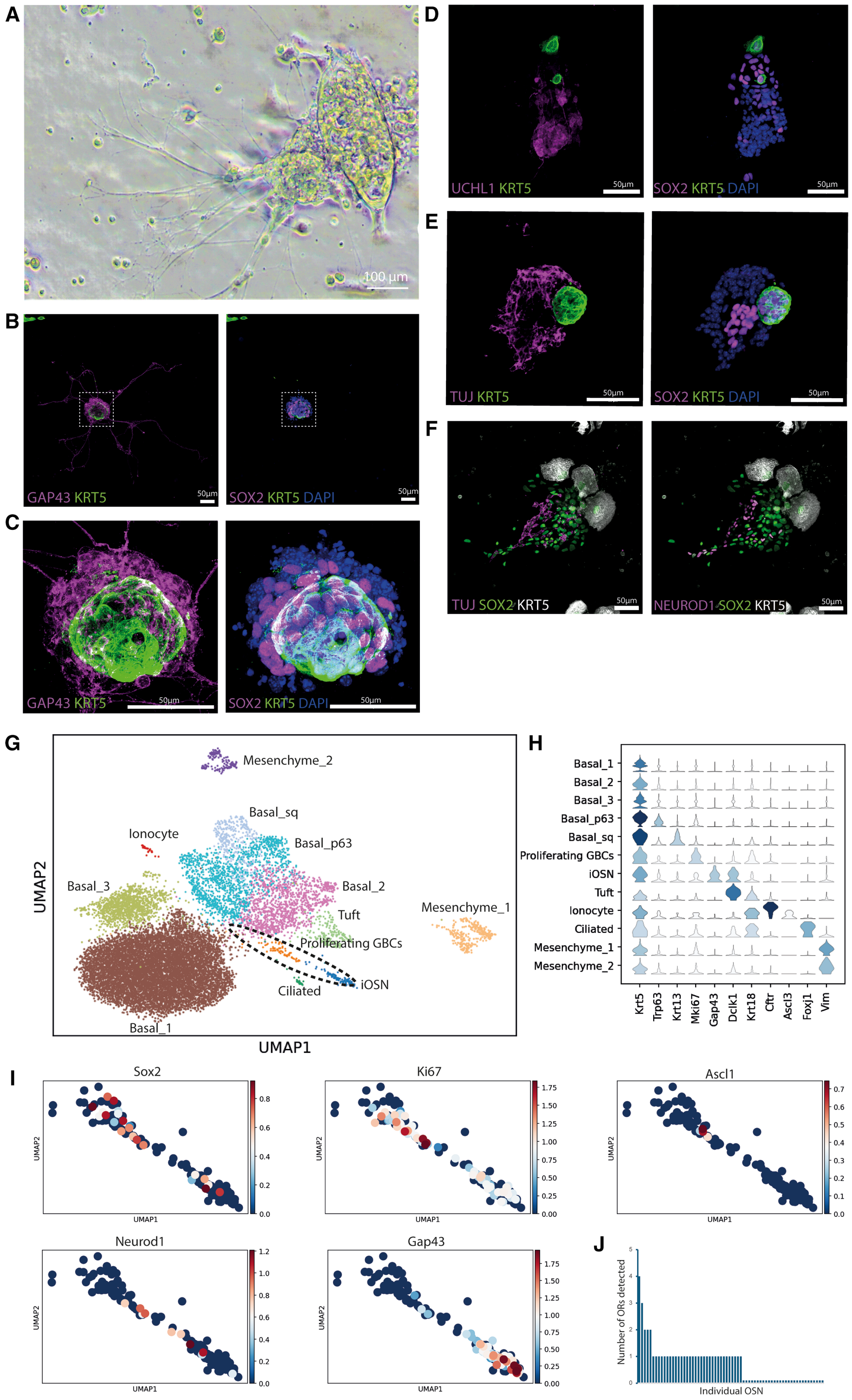

(legend on next page)

CellPress
OPEN ACCESS

basal cells (GBCs). HBCs lie directly on top of the basement membrane and are a dormant population of reserve stem cells. GBCs, on the other hand, dwell apically to HBCs and are composed of a highly heterogeneous population, including both actively proliferating progenitors and true tissue stem cells, responsible for the homeostatic maintenance of the tissue.[6] It has been shown that the human OE also contains these two stem cell populations and can continually generate OSNs throughout life.[7] However, injuries such as viral infection, exposure to environmental toxins, or even aging itself can compromise this neurogenic process.[8–13] Indeed, our sense of smell significantly diminishes with age, likely through the gradual loss of OSNs and GBCs.[8,10,14,15] No clinically validated, molecularly based therapeutic intervention has yet been developed to treat olfactory dysfunction outside of smell loss due to chronic rhinosinusitis.[16] This problem has, in part, been due to the lack of a robust *in vitro* model of the OE that preserves its cellular and architectural complexities.

In other tissues, the development of tissue-faithful organoids that can mimic *in vivo* composition and structures has been the catalyst driving significant contributions to our understanding of tissue-specific organization, development, and cell-cell communications.[17] In the OE, prior work has explored the explant culture of embryonic tissue, purified embryonic stem cell cultures, or homogeneous cultures of specific OE stem cells. In these cases, the complexity of adult neurogenesis is either not modeled or not fully recapitulated.[18–25] More recently, organoid models from embryonic and adult OE tissue have emerged.[26,27] These cases require either fluorescence-activated cell sorting (FACS) of specific adult stem cells using transgenic animal reporters or difficult dissection of embryonic tissue and multiple steps for axon growth. Here, we establish a highly robust and facile organoid model of the adult murine OE that requires only standard adult tissue dissection without the need for transgenic animals or FACS purification. Our model recapitulates adult neurogenesis *in vitro,* generating immature OSNs that express all of the components necessary for odorant sensing and are on the cusp of maturing into OMP+ OSNs. In contrast to standard organoid cultures grown in full suspension, our organoids are fully attached to the growth substrate, allowing the development of axonal projections *in situ*. Finally, we used our model to demonstrate that HBCs, despite their own quiescence, act as a necessary neurogenic niche component for GBCs.

RESULTS

Organoid characterization

Previous work has shown that LGR5+ GBCs are multipotent *in vivo*, that GBCs can be cultured *in vitro*, and finally, that LGR5+ GBCs embedded in Matrigel can be induced to form organoids.[19,20,22,26] Despite these successes, the structures that were generated in the cultures lacked the characteristic organizational structure found in the OE. We wondered whether the usual interactions between cells *in situ* can function to organize the assembly of a more complex tissue. Accordingly, instead of culturing purified GBCs, we tested whether a combination of the many cell types in the adult tissue could result in a more organized structure.

To that end, the olfactory mucosa of adult mice, comprising both OE and lamina propria, was separated from the adjacent respiratory tissue[18] and then dissociated using papain. We reasoned that the fibroblasts of the lamina propria might overtake the epithelial cells in culture, so we discarded cells that had adhered to tissue culture plastic within 1 h of plating and cultured the remainder in medium containing WNT signaling components comprised of 200 ng/mL R-spondin1, 100 ng/mL Noggin, and 50 ng/mL Wnt3a.[26,28,29]

Reanalysis of single-cell RNA sequencing (RNA-seq) datasets of dissociated olfactory tissue suggests that *bona fide* HBCs and GBCs comprise only ~4% and ~0.9%, respectively, of all dissociated cells.[30] Considering that, we optimized the seeding density of these cells to 940 cells/mm^2, or approximately 75,000 cells/well of an 8-well chamber slide, so that the organoid number was maximized while maintaining distinct structures. Overseeded cultures exhibited merging of organoids, making it challenging to quantify individual organoids (Figure S1A). We confirmed that the vast majority of cells that are dissociated are damaged mature sustentacular cells, OSNs, and duct/gland cells that all die and are cleared from the culture within the first 2 days (Figure S1B).

By day 6 in culture, neurite-like projections emanated from newly formed 3D structures that were attached to the bottom of the well (Figure 1A). Given the appearance of these projections, we immunostained for canonical markers of OSNs (GAP43, UCHL1, and TUBB3A/Tuj1) as well as proteins that are characteristic of GBCs (SOX2 and NEUROD1) and HBCs (KRT5) (Figures 1B–1F). The immunostaining demonstrates that organoids contain HBC-like cells (KRT5+/SOX2+) in close association with upstream GBC-like cells (KRT5−/SOX2+), neuronally committed/neuron-producing downstream NEUROD1+ GBC-like cells (KRT5−/SOX2+/NEUROD1+), and immature OSN (iOSN)-like cells (KRT5−/SOX2−/Tuj+/GAP43+/UCHL1+). Indeed, all projections emanating from the organoids were positive for Tuj1, UCHL1, and GAP43, indicating that they were indeed neurites extended from neuron somata. Continuous neurogenesis and OSN survival were observed for at least 2 weeks, though we considered day 7 to be a reasonable time point for characterization, as no new organoids formed afterward. Thus, on day 7, we obtained an average of 30.5 organoids per well,

Figure 1. Organoid characterization
(A) Representative bright-field image of an organoid on the seventh day of culture.
(B and C) Immunostaining of a representative organoid for iOSNs (Gap43), HBCs (KRT5), and GBCs (SOX2+, KRT5-). (C) shows high-magnification images of (B).
(D–F) Representative immunostaining of organoids for other neurogenic markers.
(G) UMAP representation of single-cell RNA-seq of the organoid culture.
(H) Stacked violin plots of key marker genes allowing cluster annotation in log scale.
(I) Focused UMAP expression plots in the dotted area shown in (G), plotted in log-scale, representing the neurogenic cascade found in the organoids.
(J) Histogram plot of the number of distinct ORs found in single cells identified as OSNs based on UCHL1, GAP43, and TUBB3.

with each organoid containing an average of 48.8 Tuj1+ OSNs (Figures S1B and S1C).

Olfactory organoids recapitulate adult neurogenesis and form iOSNs

To characterize our cultures and confirm our putative cellular identifications, we performed single-cell RNA-seq of our organoid cultures and identified 12 distinct cell clusters (Figure 1G). Unsurprisingly, mesenchymal cells were found in the cultures, along with several distinct *Krt5*+ basal cell populations. Of these, one in particular expressed high levels of the canonical basal cell marker *Trp63*, which we label here as "Trp63 basal." Another basal cell cluster was distinguished by high levels of the squamous marker *Krt13*, which we term "squamous basal." Of note, despite performing stringent microdissection of OE and explicitly removing the respiratory epithelium, we still recovered ciliated cells (*Foxj1* and *Krt18*), suggesting that either they were contaminants through the dissection process or that olfactory stem cells demonstrated plasticity and generated these in our culture model, as they sometimes do *in vivo*[31] (Figure 1H). The latter would be consistent with known reports of respiratory metaplasia found deep within the OE after severe injury or with age.[8,15,31] Encouragingly, we also found several clusters of cells that recapitulated the stepwise path of adult neurogenesis found in the OE.[6] We used partition-based graph abstraction to computationally infer cell fate trajectory on these cells and confirmed that these *in vitro*-cultured cells matched what is described in published data: an actively dividing, *Sox2*+, *Ki67*+, multipotent stem GBC population transitioning into neuronally specified *Ascl1*+ GBCs, evolving into *Neurod1*+ neuronal progenitor GBCs, and finally culminating in a *Gap43*+ iOSN (Figures 1I and S2A). More than half of the profiled iOSN (37 of 67) expressed a detectable olfactory receptor. Although up to four receptors were detected in a single OSN, most of these neurons expressed only one receptor (Figure 1J). In total, 39 distinct receptors were identified. Notably, these cells did not express *Omp*, a key marker of mature OSNs. On the other hand, we were able to detect the expression of both *Ezh2* and *Kdm1a* (*Lsd1*), both epigenetic regulators implicated in olfactory receptor choice, as well as key ion channels required for OSN function, including *Ano2* and *Scn9a*. Since truly immature OSNs are known to express multiple ORs while fully mature OSNs generally express one OR while also expressing *Omp* in addition to the key ion channels described above, we concluded that the iOSNs formed in this organoid culture are at the cusp of expressing Omp and becoming *bona fide* mature OSNs.[32–35] Given the significant role the olfactory bulb plays in mature OSN (mOSN) survival and lifespan and that olfactory bulbectomy results in the massive apoptosis of mOSN,[4,36–40] we hypothesize that the lack of an olfactory bulb and its associated trophic factors contributed to the lack of mOSNs in our culture (Figure S2B). Thus, besides these mature OSNs, our organoid model contains the full spectrum of cells within the adult neurogenic path in the OE, including quiescent HBCs and active GBCs.

Optimization of olfactory organoid cultures

As both multipotent HBCs and GBCs are found in adult OE tissue, the organoids that form in the mixed culture possibly arose from one or both cell populations. It is known that GBC numbers increase during regeneration and reduce with age.[8,10,15,41] Thus, we tried to optimize the efficiency of organoid generation in our model using two different approaches: using lesioned OE or adolescent OE as the starting material.

For the first approach, we directly injured the OE through a single intraperitoneal injection of methimazole to stimulate the expansion of multipotent GBC stem cells.[9,42–44] To contextualize the results, we first analyzed the dynamics of the stem and progenitor cell populations following methimazole injury using immunofluorescence (Figure S3) and RT-qPCR of a time course following injury (Figures S4A–S4C).

We confirmed that methimazole injury ablates nearly all mature cells of the OE by day 1 post injection. Surprisingly, BSND+ ionocytes (also known as IP3R3+ microvillar cells[30,45]) survived injury and were found interspersed with the remaining HBCs and GBCs even on day 1 post methimazole. As expected, based on previous experience using similar injury models, HBCs were the primary proliferating cell population found in the tissue until day 5 post injury, when a basal monolayer of HBCs was reinstated in the OE; at this time point, SOX2+ GBCs are the primary proliferating population. Between 4 and 5 days post methimazole injury, there was higher expression *in vivo* of genes for multipotent stem cell (*Lgr5*, *c-kit*, and *Trp63*), neurogenic markers (*Ascl1* and *Neurog1*), and the non-neurogenic marker of ionocytes, *Foxi1* (Figures S3 and S4A–S4C). GBCs progressed along previously reported trajectories, with neuronally fated ASCL1+ and NEUROD1+ GBCs emerging around day 3 post injury *in vivo* and reaching a maximum by day 5 post injury.[6] This sequential emergence of cell types *in vivo* corroborated the cell state transition results revealed by the single-cell analysis within our organoid cultures.

From paired lesioned littermates, we harvested OE, cultured dissociated cells at the time points shown above, and evaluated them for the subsequent formation of Tuj1+ OSNs. We found that, when OE is harvested and cultured 4 and 5 days after injury, significantly higher numbers of Tuj1+ OSNs were generated when compared with the unlesioned control (Figure S3D, $p < 0.05$).

For the second approach, we tested whether age had an impact on *in vitro* neurogenesis, as it has been shown that GBCs, including LGR5+ GBCs, are more numerous in younger mice and that aged mice have less neurogenic capacity.[8,20] We found that, indeed, 3-week-old donor mice generated significantly more Tuj1+ OSNs than either 24- or 52-week-old donors. At 6 weeks and beyond, no statistically significant differences were detected (Figures S4E and S4F).

HBCs remain quiescent within organoids

Though post-methimazole injury conditions and 3-week-old mice produced significantly more Tuj1+ OSNs, we elected to use uninjured adult donor animals for the remainder of the experiments to more closely model normal epithelial homeostasis. Given the highly suggestive morphology of the HBC "cap" found in many of these organoids, the various HBC-like populations identified in the single-cell RNA sequencing of our cultures and previously published results using only GBCs,[26] we wondered whether HBCs harvested from the donor tissue significantly contributed to the organoids. To

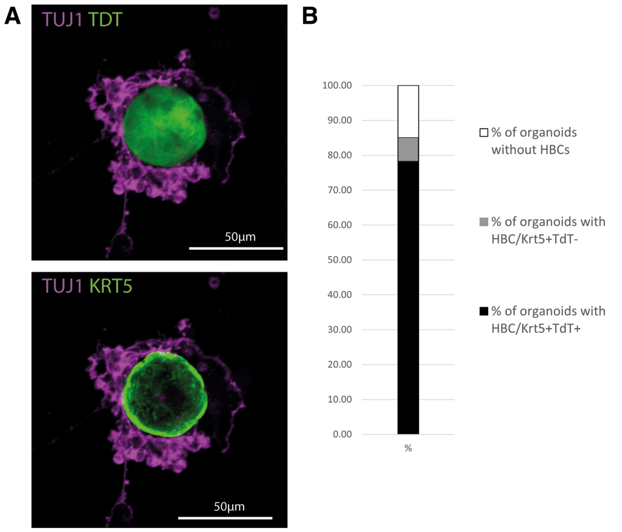

Figure 2. HBC lineage trace

(A) Representative immunostaining of an organoid derived from a KRT5-CreER;LSL-TdTomato lineage trace mouse that was tamoxifen-induced *in vivo* 1 week prior to culture. Organoids were fixed and stained 1 week after culture.

(B) Percentage of organoids with HBCs labeled and unlabeled with TdTomato. *N* = 9 distinct experiments performed on separate days using different mice.

test this hypothesis, KRT5-CreER;LSL-TdTomato (TdT) mice were injected with tamoxifen prior to tissue harvest to lineage trace pre-existing HBCs in the starting material and visualize their contribution to the cultured organoids. Recombination efficiency was nearly complete, at 88% (SD = 0.08%), similar to what we have demonstrated and imaged previously.[18,46,47] While HBCs were again found in the vast majority of organoids (85%), lineage-labeled, TdT+ HBCs strikingly did not contribute to the neuronal cell populations and remained as HBCs *in vitro*, consistently mirroring their normal behavior *in vivo* under homeostasis (Figure 2). Indeed, during all of our experiments (n = 9 distinct culture experiments), we observed only a single HBC-derived cell that had differentiated into a non-KRT5+ cell. Finally, we found little or no evidence for dedifferentiation of GBCs into HBCs under these conditions, as 92% of all KRT5+ HBCs were derived from lineage-labeled HBCs.

Quiescent HBCs can function as a neurogenic niche for GBCs

Despite the complete lack of HBC differentiation in the organoids, the consistent appearance of HBCs in the majority of organoids led us to hypothesize that they could act as a niche or play some role in the formation of these organoids. To test for a role in the formation of the neurogenic organoids, we mixed purified populations of HBCs with purified populations of neuronally specified GBCs. HBCs were isolated using tamoxifen-induced KRT5-CreER;LSL-TdTomato animals, while ASCL1+ GBCs and NEUROG1+ GBCs were isolated based on ASCL1-TdTomato and Neurog1-eGFP signals, respectively. As expected, HBCs, when cultured alone, remained HBCs and did not form any Tuj1 + cells under these conditions. ASCL1+ GBCs alone produced only a handful of Tuj1+ OSNs, with most cells failing even to survive. Surprisingly, in the presence of HBCs, ASCL1+ GBCs produced significantly more neurons, as measured by total Tuj1 immunofluorescence than either cell population alone ($p < 0.05$; Figure 4F). Interestingly, purified Neurog1+ GBCs were incapable of proliferating or surviving with or without HBC co-culture (Figures 3D and 3E). Their limited survival capacity when cultured alone is consistent with their status as a more downstream, limited neuronal progenitor population that is only a day or two away from becoming terminally differentiated neurons,[48–50] and we speculate that they need a fully intact niche for survival.

These experiments, utilizing mixtures of purified cells as starting material, support the hypothesis that HBCs play a role in the formation of organoids. To determine whether this was mediated through physical contact or secreted factors, we used a Transwell system to permit only the free flow of medium and soluble factors through the membrane while preventing contact between the different cell types. Indeed, other reports have also demonstrated the inadequacy of HBCs alone in producing organoids in culture.[51] We seeded dissociated OE on the top layer of the Transwell membrane and confirmed that this Transwell system supported organoid growth and neuronal differentiation (Figures 4A and 4D). In comparison, when we depleted HBCs from the seeded cells using FACS, we observed no differentiation or survival (Figures 4B and 4E) However, when HBCs were plated in the bottom chamber of the Transwell, we saw a modest rescue of differentiation and survival but not a complete recapitulation of the organoid structures formed when they were physically in contact with each other (Figures 4C and 4F). Taken altogether, this suggests that HBCs maintain a niche for neurogenesis through both physical interaction and soluble factors released into the medium. To control for differences in culture conditions, we depleted HBCs from dissociated, uninjured OE using FACS purification, plated the remainder into standard chamber wells, and confirmed that, indeed, organoids and neuronal differentiation occurred only in the presence of the HBCs (Figures S5A–S5C). Given that injury and regeneration conditions have been shown to induce plasticity in epithelial systems, including the OE, we tested whether regenerating OE required HBCs to function as a niche. We injured donor mice with a single intraperitoneal injection of methimazole and harvested cells 4 days after injection, the earliest point when HBCs and GBCs were easily distinguishable (Figure S3). Again, we found that the depletion of HBCs prevented significant neurogenesis from the remaining cell populations (Figures S5D and S5E). Thus, in this organoid culture system, HBCs form a niche that regulates GBC survival, maintenance, and neuronal differentiation in both a cell contact and secreted manner.

In silico modeling of the HBC-GBC stem cell niche

Finally, we sought to identify the potential niche factors that HBCs may be providing GBCs through cell-cell interaction predictive algorithms. Our single-cell RNA-seq data did not contain enough cells for robust predictions, so we elected to use a pre-existing single-cell RNA-seq dataset of uninjured mouse OE that was highly robust and contained abundant cells.[30] We computationally isolated cells in the direct neuronal olfactory lineage

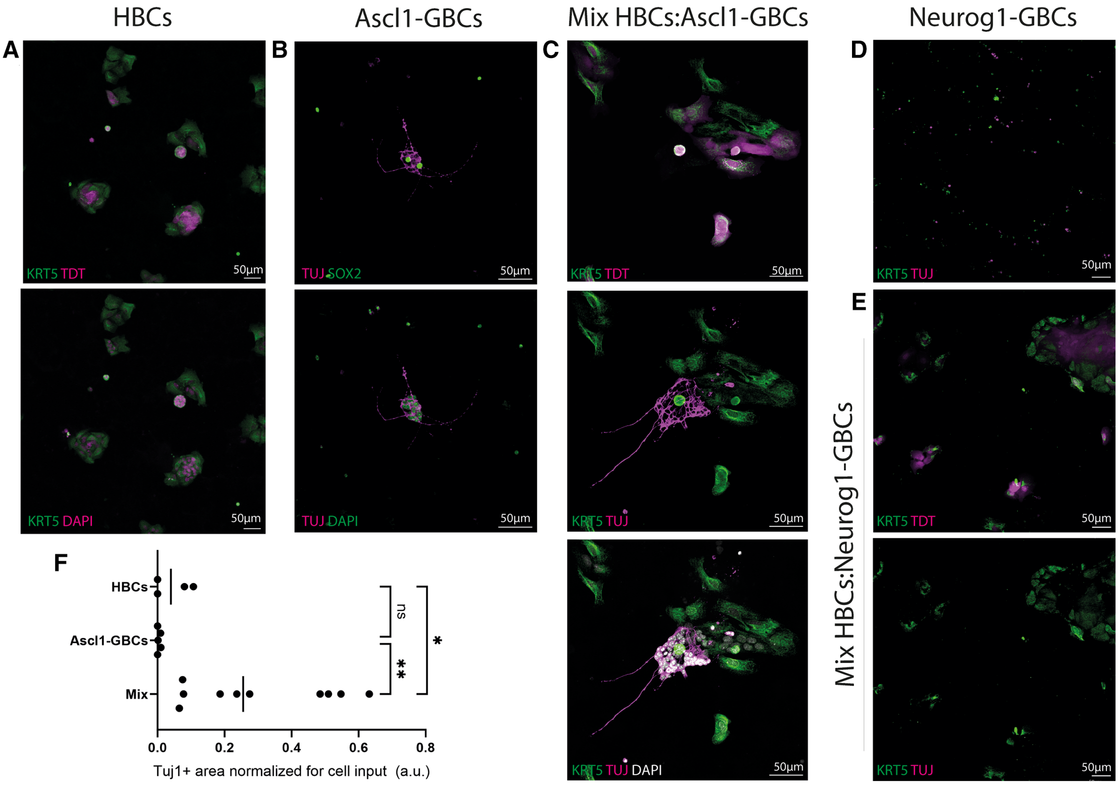

Figure 3. Reconstitution experiments

(A–E) Immunostaining of HBCs cultured alone (A), Ascl-1-GBCs alone (B), HBCs with ASCL1-GBCs (C); Neurog1-GBCs alone (D), and HBCs and Neurog1-GBCs (E).

(F) Quantification of the Tuj1+ area normalized for cell input (a.u.) of HBCs, ASCL1-GBCs, and the two together. ANOVA comparison, $p = 0.0057$, *$p < 0.05$, **$p < 0.01$. Dots represent distinct biological inputs plated spread over 3 different days using different mice (HBCs = 4, ASCL1-GBCs = 5, mixture = 10).

(HBCs, GBCs, iOSNs, and mOSNs) and, instead of choosing individual algorithms that can yield highly variable results, we performed aggregate LIANA+ analysis on this subset. In this analysis, the results of many different predictive algorithms are aggregated and scored for specificity and magnitude.[52,53] Not surprisingly, we found that HBCs and GBCs had many predicted interactions that included Notch, Wnt, Semaphorin, and Neuregulin pathways (Figures 4H and 4I), suggesting that the two stem cell populations form a bi-directional niche. Three of the most significant and highly expressed signaling pairs were Midkine-Syndecan4, Semaphorin3d-Neuropilin2/PlexinA3, and Kitl-Kit (Figure 4J). Midkine/Syndecan signaling has been shown to promote the maintenance and proliferation of neural progenitor cells,[54] and Semaphorin/Neuropilin signaling has been implicated in olfactory regeneration and axon sorting.[55–57] KIT signaling has long been known to regulate stem cell behavior including neuronal stem cells.[58–60] Most intriguingly, in the olfactory system, it has been shown elegantly with genetic lineage tracing that c-Kit knockout and small-molecule inhibitors that active KIT signaling appear to promote self-renewal over terminal differentiation in GBC cultures.[19,61,62] In sum, the data support a model where HBCs and GBCs together form a niche that supports adult neurogenesis that may include active Midkine, Semaphorin, and KIT signaling.

DISCUSSION

The OE, beyond its critical role in maintaining our sense of smell, is a robust source of two distinct neural stem cell populations. One exhibits all of the markers of dormant reserve epithelial basal stem cells, while the other is heterogeneous and resembles canonical neural stem cells found in the CNS.[6] Understanding the complex relationship between these two stem cell populations and what maintains them when most other neural stem cells disappear from the CNS and peripheral nervous system during development would not only impact our ability to design therapies for the loss of smell but could also be a key to understanding and harnessing adult neurogenesis. To this end, many groups have attempted to create *in vitro* models of the OE, varying in complexity from isolated attached cells to complex structures in suspension and even to the OE as a whole. Embryonic tissue has been shown to grow *ex vivo*, capable of generating OSNs, while we and others have shown that HBCs and GBCs can be cultured individually.[18–20,50] Even more recently, groups have generated organoid cultures of the OE using selected GBC populations.[26,41,63] These models have significant advantages compared to studying the OE *in vivo*. However, they all possess critical weaknesses that hamper their use in studying the stem cell dynamics found within the OE—they require hard-to-get,

CellPress
OPEN ACCESS

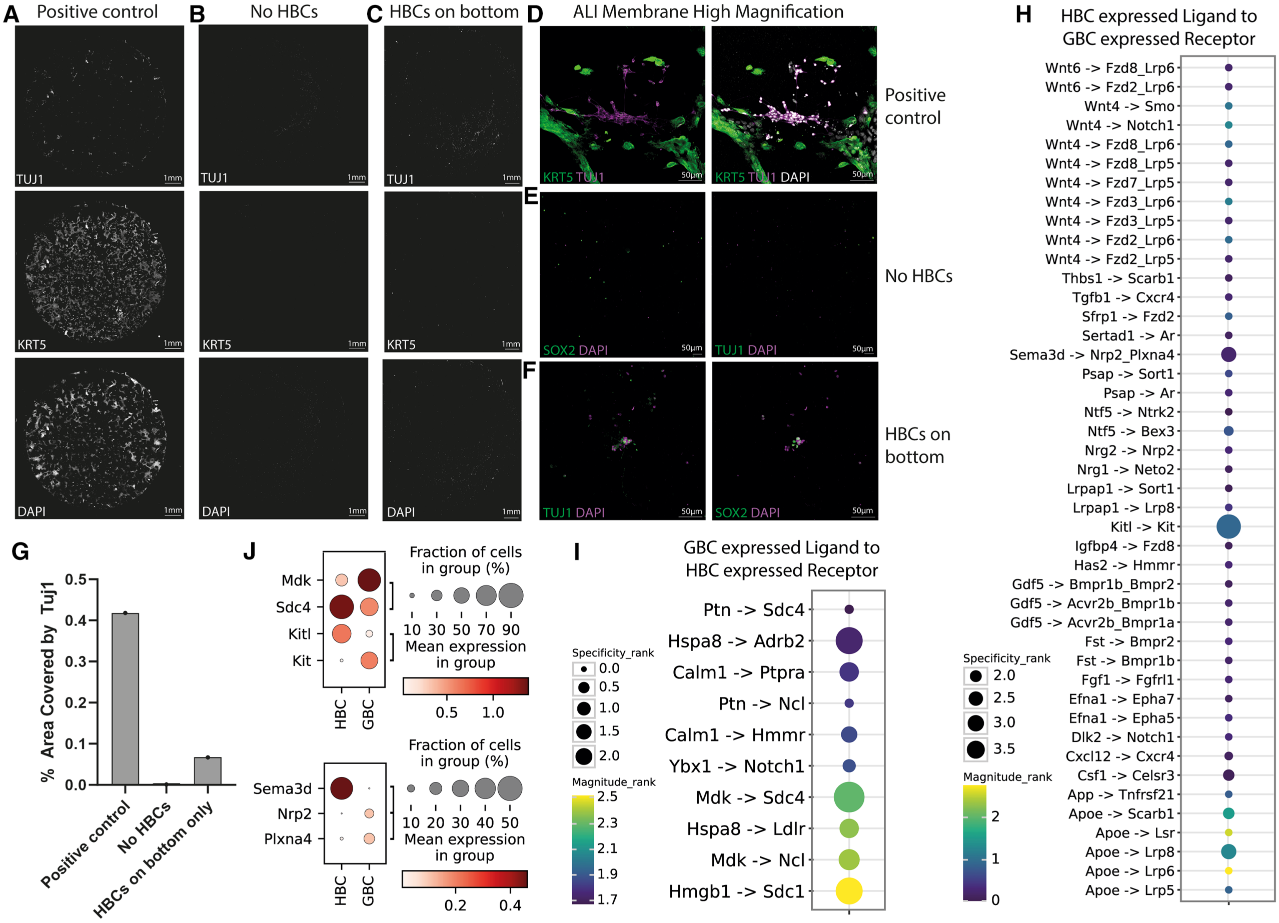

Figure 4. Secretion vs. contact dependency experiments

(A–C) Immunostaining of entire Transwell membranes of positive controls (dissociated cells that were run through the FACS process and reconstituted at the original ratios of HBCs and the negative population) (A), HBC depleted (B), and HBCs seeded on the bottom chamber (C). Contrast was increased significantly to facilitate visualization.

(D–F) High-magnification images of regions shown in (A)–(C).

(G) Quantification of the percentage of area covered by Tuj1 in the Transwells. Under HBC-depleted conditions, note the near-complete lack of detected signal. Experiments were done on two distinct days, FACS purified from a pool of mice. Two technical replicates each.

(H) Dotplot representations of LIANA+ aggregate predictions of HBC signals being received by GBCs.

(I) Similar dotplot representations of predicted GBC signals being received by HBCs.

(H and I) The size of the dot represents the specificity of the interaction, while the color marks the magnitude.

(J) Dotplot representation of the expression of the top 3 predicted interactions between HBCs and GBCs. Plots are split for visual clarity due to differences in scale, where the size of the dot represents the fraction of the cells expressing the gene, and the color intensity represents the mean expression.

limited tissue samples, cannot expand significantly or only expand purified populations that do not exhibit high differentiation efficiencies, create disorganized or random swaths of cells, or require sorting of rare starting populations.

Here, we describe a 3D organoid culture that performs regardless of the donor animals' age past adulthood, requires no purification of starting material, recapitulates adult neurogenesis until the iOSN stage, and self-organizes, even generating outward axonal projections. We specifically highlight that the use of adherent plastic, combined with low-percentage extracellular matrix, are critical for these structures. Notably, however, the model does not contain sustentacular or duct/gland cells, as WNT agonism is used explicitly to push neurogenesis, nor does it contain mature OSNs. Thus, we propose that this organoid model is most useful for studying neurogenic stem cell dynamics and neurogenesis. We hypothesize that the lack of mOSNs is likely due to the lack of the olfactory bulb, which has been shown to provide signals important for maturation and survival.[4,36–40] In addition, it is possible that other signals, including active odorant binding[64,65] or factors expressed by the lamina propria, including olfactory ensheathing cells,[66] contribute to maturation.

Previous work has demonstrated that, during the extremes of life, either at early development or near the end of life, with extreme dietary restriction of retinoic acid, HBCs can be active stem cells.[67,68] However, during much of normal adult life,

HBCs have been shown to be generally quiescent in the absence of injury.[44,47,69] Our cultures appear to recapitulate HBC stem cell quiescence, though we were surprised to find that HBCs were critical for organoid formation by acting as a niche for the GBCs. Our reconstitution assays led to the identification of both soluble factors and direct cell-cell interactions as components of this neurogenic niche. We find that soluble factors released by HBCs can improve neurogenesis, though it cannot completely replace direct contact. This effect can likely be seen in the small percentage of organoids that altogether lack HBCs (Figure 2B). Past work studying age-associated anosmia in both murine and human tissue suggests that GBCs exhaust first, resulting in an aneurogenic/aneuronal OE that may progress to respiratory metaplasia, potentially arising from the retained HBCs.[8,10,14,15,41] Our data suggest that HBCs provide a niche for neurogenic GBCs, leading to our hypothesis that GBCs may, in turn, provide a niche for HBCs to remain neurogenic as well. While elucidating these mechanisms would be difficult *in vivo*, our new *in vitro* model will enable the field to perform not only complex genetic and small-molecule manipulations but also reconstitution assays.

Extending this platform to human brush biopsies would allow for toxicity testing, disease modeling, aging, and testing treatments, though further work will be needed to extend this model to human tissue, such as improving the efficiency and passaging of organoid culture, identifying the conditions necessary for stable maturation of OSNs, and identifying conditions that support co-induction of the other cell types of the OE. In particular, while several studies have attempted elegant methods to identify the key trophic factors the olfactory bulb expresses to enable the maturation and survival of OSNs, definitive factors have not been identified.[39,40,70–72] We propose that our new culture model could serve as the ideal screening platform for such an endeavor. Our single-cell RNA-seq data also demonstrate a high level of heterogeneity in the KRT5+ basal cells found in our culture. Whether this represents a previously unappreciated level of true heterogeneity that is made detectable in tissue culture or is a tissue culture artifact remains to be seen.

Using cell-cell interaction analysis, we computationally identified possible pathways through which GBCs and HBCs together form a niche for adult neurogenesis, including Midkine-Syndecan4, Semaphorin3d-Neuropilin2/PlexinA3, and Kitl-Kit. While our model and bioinformatic analysis suggests that these signaling molecules could regulate neural progenitors and adult neurogenesis, such an analysis is limited in its predictive capabilities. Further wet-lab investigation is required to validate the biological finding *in vitro* and *in vivo*.

Limitations of the study

We report a 3D organoid model of the murine OE that is cultured on top of an adherent substrate using primary mouse olfactory cells from adult animals. Eventually, our cultures exhibited neural exhaustion, at least partially caused by the lack of an olfactory bulb and the supporting factors that it secretes to promote maturation and survival of OSNs. It will be necessary to identify methods of promoting OSN survival and maturation and better methods of passaging or expanding the organoids. Due to our focus on neurogenesis and pro-neurogenic culture conditions, we skewed differentiation away from other cell types, such as sustentacular or duct/gland cells. An important improvement would be to identify a more neutral or stepwise differentiation protocol to allow non-neuronal cell differentiation. Finally, our study was limited to mouse cells, and a critical next step will be to optimize this methodology to support human cell growth, which is not directly supported with this protocol, likely not only due to differences in the needed supplements for human cells but also because human samples tend to be far more contaminated with respiratory epithelium, which overtakes the culture.

RESOURCE AVAILABILITY

Lead contact

Requests for further information and resources should be directed to the lead contact, Brian Lin (brian.lin@tufts.edu).

Materials availability

This study did not generate new unique reagents.

Data and code availability

- Single-cell RNA-seq FASTQ files and processed read counts were deposited at GEO (GEO: GSE257536). All other source data are included in the supplemental information.
- This study did not generate original code.
- Any additional information required to re-analyze the data reported in this paper is available from the lead contact upon request.

ACKNOWLEDGMENTS

We thank Po Kwok-Tse for her extraordinary technical assistance and Woochan Jang for his support and assistance. We also thank Albert Tai, Allen Parmelee, and Stephen Kwok of the David Thorley-Lawson Memorial Flow Cytometry Core Facility (NIH S10OD032201) for assistance. This study was supported by the National Institutes of Health (projects NIDCD R21DC018681-01 and R01DC017869-03), in part by the Coordenação de Aperfeiçoamento de Pessoal de Nível Superior – Brasil (CAPES) (finance code 001), and funding from the German Research Foundation (DFG) under the Walter Benjamin Program and by the Fritz Thyssen Foundation.

AUTHOR CONTRIBUTIONS

J.G.G. and B.L. designed the experiments. J.G.G., C.A.H., A.D., and B.L. conducted the experiments. J.E.S. provided funding; J.G., C.A.H., A.D., V.H., J.S., E.H.H., M.A.F., and B.L. reviewed the data and wrote the paper.

DECLARATION OF INTERESTS

J.E.S. is a co-founder of Rhino Therapeutics. E.H.H. and B.L. are consultants for Rhino Therapeutics. B.L. and C.A.H. are co-founders of Cellsor Inc.

STAR★METHODS

Detailed methods are provided in the online version of this paper and include the following:

- KEY RESOURCES TABLE
- EXPERIMENTAL MODEL AND STUDY PARTICIPANT DETAILS
- METHOD DETAILS
  - Tissue processing for cell culture
  - Tissue processing for sections
  - Bulk RNA isolation
  - Fluorescence-activated cell sorting (FACS)
  - Cell culture

- ○ Growth media
- ○ Single-cell RNAseq
- ○ Immunofluorescence
- QUANTIFICATION AND STATISTICAL ANALYSIS

SUPPLEMENTAL INFORMATION

Supplemental information can be found online at https://doi.org/10.1016/j.crmeth.2025.101055.

Received: January 21, 2025
Revised: April 4, 2025
Accepted: April 30, 2025
Published: May 28, 2025

REFERENCES

1. Graziadei, P.P., and Graziadei, G.A. (1979). Neurogenesis and neuron regeneration in the olfactory system of mammals. I. Morphological aspects of differentiation and structural organization of the olfactory sensory neurons. J. Neurocytol. *8*, 1–18.
2. Gaun, V., Martens, J.R., and Schwob, J.E. (2022). Lifespan of mature olfactory sensory neurons varies with location in the mouse olfactory epithelium and age of the animal. J. Comp. Neurol. *530*, 2238–2251.
3. Hinds, J.W., Hinds, P.L., and McNelly, N.A. (1984). An autoradiographic study of the mouse olfactory epithelium: evidence for long-lived receptors. Anat. Rec. *210*, 375–383.
4. Holcomb, J.D., Mumm, J.S., and Calof, A.L. (1995). Apoptosis in the neuronal lineage of the mouse olfactory epithelium: regulation in vivo and in vitro. Dev. Biol. *172*, 307–323.
5. Holl, A.-M. (2018). Survival of mature mouse olfactory sensory neurons labeled genetically perinatally. Mol. Cell. Neurosci. *88*, 258–269.
6. Schwob, J.E., Jang, W., Holbrook, E.H., Lin, B., Herrick, D.B., Peterson, J. N., and Hewitt Coleman, J. (2017). Stem and progenitor cells of the mammalian olfactory epithelium: Taking poietic license. J. Comp. Neurol. *525*, 1034–1054.
7. Durante, M.A., Kurtenbach, S., Sargi, Z.B., Harbour, J.W., Choi, R., Kurtenbach, S., Goss, G.M., Matsunami, H., and Goldstein, B.J. (2020). Single-cell analysis of olfactory neurogenesis and differentiation in adult humans. Nat. Neurosci. *23*, 323–326.
8. Child, K.M., Herrick, D.B., Schwob, J.E., Holbrook, E.H., and Jang, W. (2018). The Neuroregenerative Capacity of Olfactory Stem Cells Is Not Limitless: Implications for Aging. J. Neurosci. *38*, 6806–6824.
9. Håglin, S., Bohm, S., and Berghard, A. (2021). Single or Repeated Ablation of Mouse Olfactory Epithelium by Methimazole. Bio Protoc *11*, e3983.
10. Oliva, A.D., Gupta, R., Issa, K., Abi Hachem, R., Jang, D.W., Wellford, S.A., Moseman, E.A., Matsunami, H., and Goldstein, B.J. (2022). Aging-related olfactory loss is associated with olfactory stem cell transcriptional alterations in humans. J. Clin. Investig. *132*, e155506. https://doi.org/10.1172/JCI155506.
11. Doty, R.L. (1989). Influence of age and age-related diseases on olfactory function. Ann. N. Y. Acad. Sci. *561*, 76–86.
12. Upadhyay, U.D., and Holbrook, E.H. (2004). Olfactory loss as a result of toxic exposure. Otolaryngol. Clin. North Am. *37*, 1185–1207.
13. Hummel, T., Whitcroft, K.L., Andrews, P., Altundag, A., Cinghi, C., Costanzo, R.M., Damm, M., Frasnelli, J., Gudziol, H., Gupta, N., et al. (2017). Position paper on olfactory dysfunction. Rhinol Suppl. *54*, 1–30.
14. Holbrook, E.H., Wu, E., Curry, W.T., Lin, D.T., and Schwob, J.E. (2011). Immunohistochemical characterization of human olfactory tissue. Laryngoscope *121*, 1687–1701.
15. Fitzek, M., Patel, P.K., Solomon, P.D., Lin, B., Hummel, T., Schwob, J.E., and Holbrook, E.H. (2022). Integrated age-related immunohistological changes occur in human olfactory epithelium and olfactory bulb. J. Comp. Neurol. *530*, 2154–2175.
16. Jafari, A., and Holbrook, E.H. (2022). Therapies for olfactory dysfunction - an update. Curr. Allergy Asthma Rep. *22*, 21–28.
17. Qian, X., Song, H., and Ming, G.-L. (2019). Brain organoids: advances, applications and challenges. Development *146*, dev166074. https://doi.org/10.1242/dev.166074.
18. Peterson, J., Lin, B., Barrios-Camacho, C.M., Herrick, D.B., Holbrook, E. H., Jang, W., Coleman, J.H., and Schwob, J.E. (2019). Activating a Reserve Neural Stem Cell Population In Vitro Enables Engraftment and Multipotency after Transplantation. Stem Cell Rep. *12*, 680–695.
19. Goldstein, B.J., Goss, G.M., Choi, R., Saur, D., Seidler, B., Hare, J.M., and Chaudhari, N. (2016). Contribution of Polycomb group proteins to olfactory basal stem cell self-renewal in a novel c-KIT+ culture model and in vivo. Development *143*, 4394–4404.
20. Chen, M., Tian, S., Yang, X., Lane, A.P., Reed, R.R., and Liu, H. (2014). Wnt-Responsive Lgr5+ Globose Basal Cells Function as Multipotent Olfactory Epithelium Progenitor Cells. J. Neurosci. *34*, 8268–8276.
21. DeHamer, M.K., Guevara, J.L., Hannon, K., Olwin, B.B., and Calof, A.L. (1994). Genesis of olfactory receptor neurons in vitro: regulation of progenitor cell divisions by fibroblast growth factors. Neuron *13*, 1083–1097.
22. Kurtenbach, S., Goss, G.M., Goncalves, S., Choi, R., Hare, J.M., Chaudhari, N., and Goldstein, B.J. (2019). Cell-Based Therapy Restores Olfactory Function in an Inducible Model of Hyposmia. Stem Cell Rep. *12*, 1354–1365.
23. Illing, N., Boolay, S., Siwoski, J.S., Casper, D., Lucero, M.T., and Roskams, A.J. (2002). Conditionally immortalized clonal cell lines from the mouse olfactory placode differentiate into olfactory receptor neurons. Mol. Cell. Neurosci. *20*, 225–243.
24. Gonzales, F., Farbman, A.I., and Gesteland, R.C. (1985). Cell and explant culture of olfactory chemoreceptor cells. J. Neurosci. Methods *14*, 77–90.
25. Goetze, B., Breer, H., and Strotmann, J. (2002). A long-term culture system for olfactory explants with intrinsically fluorescent cell populations. Chem. Senses *27*, 817–824.
26. Ren, W., Wang, L., Zhang, X., Feng, X., Zhuang, L., Jiang, N., Xu, R., Li, X., Wang, P., Sun, X., et al. (2021). Expansion of murine and human olfactory epithelium/mucosa colonies and generation of mature olfactory sensory neurons under chemically defined conditions. Theranostics *11*, 684–699.
27. Duan, X. (2025). eLife Assessment: Reconstruction of functional olfactory sensory tissue from embryonic nasal stem cells. Preprint at eLife https://doi.org/10.7554/elife.102561.1.sa3,
28. Clevers, H. (2016). Modeling Development and Disease with Organoids. Cell *165*, 1586–1597.
29. García-Posadas, L., Arranz-Valsero, I., López-García, A., Soriano-Romaní, L., and Diebold, Y. (2013). A new human primary epithelial cell culture model to study conjunctival inflammation. Investig. Ophthalmol. Vis. Sci. *54*, 7143–7152.
30. Ualiyeva, S., Lemire, E., Wong, C., Perniss, A., Boyd, A.A., Avilés, E.C., Minichetti, D.G., Maxfield, A., Roditi, R., Matsumoto, I., et al. (2024). A nasal cell atlas reveals heterogeneity of tuft cells and their role in directing olfactory stem cell proliferation. Sci. Immunol. *9*, eabq4341.
31. Xie, F., Fang, C., Schnittke, N., Schwob, J.E., and Ding, X. (2013). Mechanisms of permanent loss of olfactory receptor neurons induced by the herbicide 2,6-dichlorobenzonitrile: effects on stem cells and noninvolvement of acute induction of the inflammatory cytokine IL-6. Toxicol. Appl. Pharmacol. *272*, 598–607.
32. Coleman, J.H., Lin, B., and Schwob, J.E. (2017). Dissecting LSD1-dependent neuronal maturation in the olfactory epithelium. J. Comp. Neurol. *525*, 3391–3413.
33. Tan, L., Li, Q., and Xie, X.S. (2015). Olfactory sensory neurons transiently express multiple olfactory receptors during development. Mol. Syst. Biol. *11*, 844.
34. Huang, J.S., Kunkhyen, T., Rangel, A.N., Brechbill, T.R., Gregory, J.D., Winson-Bushby, E.D., Liu, B., Avon, J.T., Muggleton, R.J., and Cheetham,

C.E.J. (2022). Immature olfactory sensory neurons provide behaviourally relevant sensory input to the olfactory bulb. Nat. Commun. *13*, 6194.

35. Bashkirova, E.V., Klimpert, N., Pourmorady, A., Monahan, K., Campbell, C.E., Osinski, J.M., Tan, L., Schieren, I., Stecky, B., Barnea, G., et al. (2023). Opposing, spatially-determined epigenetic forces impose restrictions on stochastic olfactory receptor choice. Elife *12*, RP87445. https://doi.org/10.7554/elife.87445.1.
36. Carr, V.M., and Farbman, A.I. (1993). The dynamics of cell death in the olfactory epithelium. Exp. Neurol. *124*, 308–314.
37. Shetty, R.S., Bose, S.C., Nickell, M.D., McIntyre, J.C., Hardin, D.H., Harris, A.M., and McClintock, T.S. (2005). Transcriptional changes during neuronal death and replacement in the olfactory epithelium. Mol. Cell. Neurosci. *30*, 583–600.
38. Calof, A.L., Rim, P.C., Askins, K.J., Mumm, J.S., Gordon, M.K., Iannuzzelli, P., and Shou, J. (1998). Factors regulating neurogenesis and programmed cell death in mouse olfactory epithelium. Ann. N. Y. Acad. Sci. *855*, 226–229.
39. Schwob, J.E., Szumowski, K.E., and Stasky, A.A. (1992). Olfactory sensory neurons are trophically dependent on the olfactory bulb for their prolonged survival. J. Neurosci. *12*, 3896–3919.
40. Bauer, S., Rasika, S., Han, J., Mauduit, C., Raccurt, M., Morel, G., Jourdan, F., Benahmed, M., Moyse, E., and Patterson, P.H. (2003). Leukemia inhibitory factor is a key signal for injury-induced neurogenesis in the adult mouse olfactory epithelium. J. Neurosci. *23*, 1792–1803.
41. Li, X., Tong, M., Wang, L., Qin, Y., Yu, H., and Yu, Y. (2020). Age-Dependent Activation and Neuronal Differentiation of Lgr5+ Basal Cells in Injured Olfactory Epithelium via Notch Signaling Pathway. Front. Aging Neurosci. *12*, 602688.
42. Fletcher, R.B., Das, D., Gadye, L., Street, K.N., Baudhuin, A., Wagner, A., Cole, M.B., Flores, Q., Choi, Y.G., Yosef, N., et al. (2017). Deconstructing olfactory stem cell trajectories at single-cell resolution. Cell Stem Cell *20*, 817–830.e8.
43. Bergman, U., Ostergren, A., Gustafson, A.-L., and Brittebo, B. (2002). Differential effects of olfactory toxicants on olfactory regeneration. Arch. Toxicol. *76*, 104–112.
44. Leung, C.T., Coulombe, P.A., and Reed, R.R. (2007). Contribution of olfactory neural stem cells to tissue maintenance and regeneration. Nat. Neurosci. *10*, 720–726.
45. Montoro, D.T., Haber, A.L., Biton, M., Vinarsky, V., Lin, B., Birket, S.E., Yuan, F., Chen, S., Leung, H.M., Villoria, J., et al. (2018). A revised airway epithelial hierarchy includes CFTR-expressing ionocytes. Nature *560*, 319–324.
46. Herrick, D.B., Lin, B., Peterson, J., Schnittke, N., and Schwob, J.E. (2017). Notch1 maintains dormancy of olfactory horizontal basal cells, a reserve neural stem cell. Proc. Natl. Acad. Sci. USA *114*, E5589–E5598.
47. Schnittke, N., Herrick, D.B., Lin, B., Peterson, J., Coleman, J.H., Packard, A.I., Jang, W., and Schwob, J.E. (2015). Transcription factor p63 controls the reserve status but not the stemness of horizontal basal cells in the olfactory epithelium. Proc. Natl. Acad. Sci. USA *112*, E5068–E5077.
48. Packard, A., Giel-Moloney, M., Leiter, A., and Schwob, J.E. (2011). Progenitor cell capacity of NeuroD1-expressing globose basal cells in the mouse olfactory epithelium. J. Comp. Neurol. *519*, 3580–3596.
49. Krolewski, R.C., Packard, A., and Schwob, J.E. (2013). Global expression profiling of globose basal cells and neurogenic progression within the olfactory epithelium. J. Comp. Neurol. *521*, 833–859.
50. Krolewski, R.C., Jang, W., and Schwob, J.E. (2011). The generation of olfactory epithelial neurospheres in vitro predicts engraftment capacity following transplantation in vivo. Exp. Neurol. *229*, 308–323.
51. Jang, W., Lambropoulos, J., Woo, J.K., Peluso, C.E., and Schwob, J.E. (2008). Maintaining epitheliopoietic potency when culturing olfactory progenitors. Exp. Neurol. *214*, 25–36.
52. Dimitrov, D., Türei, D., Garrido-Rodriguez, M., Burmedi, P.L., Nagai, J.S., Boys, C., Ramirez Flores, R.O., Kim, H., Szalai, B., Costa, I.G., et al. (2022). Comparison of methods and resources for cell-cell communication inference from single-cell RNA-Seq data. Nat. Commun. *13*, 3224.
53. Dimitrov, D., Schäfer, P.S.L., Farr, E., Rodriguez-Mier, P., Lobentanzer, S., Badia-I-Mompel, P., Dugourd, A., Tanevski, J., Ramirez Flores, R.O., and Saez-Rodriguez, J. (2024). LIANA+ provides an all-in-one framework for cell-cell communication inference. Nat. Cell Biol. *26*, 1613–1622.
54. Wang, Q., Yang, L., Alexander, C., and Temple, S. (2012). The niche factor syndecan-1 regulates the maintenance and proliferation of neural progenitor cells during mammalian cortical development. PLoS One *7*, e42883.
55. Schwarting, G.A., Kostek, C., Ahmad, N., Dibble, C., Pays, L., and Püschel, A.W. (2000). Semaphorin 3A is required for guidance of olfactory axons in mice. J. Neurosci. *20*, 7691–7697.
56. Williams-Hogarth, L.C., Puche, A.C., Torrey, C., Cai, X., Song, I., Kolodkin, A.L., Shipley, M.T., and Ronnett, G.V. (2000). Expression of semaphorins in developing and regenerating olfactory epithelium. J. Comp. Neurol. *423*, 565–578.
57. Cloutier, J.F., Giger, R.J., Koentges, G., Dulac, C., Kolodkin, A.L., and Ginty, D.D. (2002). Neuropilin-2 mediates axonal fasciculation, zonal segregation, but not axonal convergence, of primary accessory olfactory neurons. Neuron *33*, 877–892.
58. Roskoski, R., Jr. (2005). Signaling by Kit protein-tyrosine kinase–the stem cell factor receptor. Biochem. Biophys. Res. Commun. *337*, 1–13.
59. Das, A.V., James, J., Zhao, X., Rahnenführer, J., and Ahmad, I. (2004). Identification of c-Kit receptor as a regulator of adult neural stem cells in the mammalian eye: interactions with Notch signaling. Dev. Biol. *273*, 87–105.
60. Zhang, S.C., and Fedoroff, S. (1999). Expression of stem cell factor and c-kit receptor in neural cells after brain injury. Acta Neuropathol. *97*, 393–398.
61. Goldstein, B.J., Goss, G.M., Hatzistergos, K.E., Rangel, E.B., Seidler, B., Saur, D., and Hare, J.M. (2015). Adult c-Kit(+) progenitor cells are necessary for maintenance and regeneration of olfactory neurons. J. Comp. Neurol. *523*, 15–31.
62. Goss, G.M., Chaudhari, N., Hare, J.M., Nwojo, R., Seidler, B., Saur, D., and Goldstein, B.J. (2016). Differentiation potential of individual olfactory c-Kit + progenitors determined via multicolor lineage tracing. Dev. Neurobiol. *76*, 241–251.
63. Wang, H., Deng, L., and Qin, X. (2023). [Development of an olfactory epithelial organoid culture system based on small molecule screening]. Sheng Wu Gong Cheng Xue Bao *39*, 318–336.
64. Watt, W.C., Sakano, H., Lee, Z.-Y., Reusch, J.E., Trinh, K., and Storm, D.R. (2004). Odorant stimulation enhances survival of olfactory sensory neurons via MAPK and CREB. Neuron *41*, 955–967.
65. Borders, A.S., Hersh, M.A., Getchell, M.L., van Rooijen, N., Cohen, D.A., Stromberg, A.J., and Getchell, T.V. (2007). Macrophage-mediated neuroprotection and neurogenesis in the olfactory epithelium. Physiol. Genomics *31*, 531–543.
66. Iwai, N., Zhou, Z., Roop, D.R., and Behringer, R.R. (2008). Horizontal basal cells are multipotent progenitors in normal and injured adult olfactory epithelium. Stem Cell. *26*, 1298–1306.
67. Håglin, S., Berghard, A., and Bohm, S. (2020). Increased retinoic acid catabolism in olfactory sensory neurons activates dormant tissue-specific stem cells and accelerates age-related metaplasia. J. Neurosci. *40*, 4116–4129.
68. Fletcher, R.B., Prasol, M.S., Estrada, J., Baudhuin, A., Vranizan, K., Choi, Y.G., and Ngai, J. (2011). p63 regulates olfactory stem cell self-renewal and differentiation. Neuron *72*, 748–759.
69. Grill, R.J., and Pixley, S.K. (1997). In vitro generation of adult rat olfactory sensory neurons and regulation of maturation by coculture with CNS tissues. J. Neurosci. *17*, 3120–3127.
70. Fischl, A.M., Heron, P.M., Stromberg, A.J., and McClintock, T.S. (2014). Activity-dependent genes in mouse olfactory sensory neurons. Chem. Senses *39*, 439–449.

71. Krammer, E.B., Lischka, M.F., and Sigmund, R. (1980). Neurotoxicity of kainic acid: evidence against an interaction with excitatory glutamate receptors in rat olfactory bulbs. Neurosci. Lett. *16*, 329–334.

72. McClintock, T.S., Khan, N., Xie, C., and Martens, J.R. (2020). Maturation of the olfactory sensory neuron and its cilia. Chem. Senses *45*, 805–822.

73. Wolf, F.A., Angerer, P., and Theis, F.J. (2018). SCANPY: large-scale single-cell gene expression data analysis. Genome Biol. *19*, 15.

74. Wolock, S.L., Lopez, R., and Klein, A.M. (2019). Scrublet: Computational Identification of Cell Doublets in Single-Cell Transcriptomic Data. Cell Syst. *8*, 281–291.e9.

75. Schindelin, J., Arganda-Carreras, I., Frise, E., Kaynig, V., Longair, M., Pietzsch, T., Preibisch, S., Rueden, C., Saalfeld, S., Schmid, B., et al. (2012). Fiji: an open-source platform for biological-image analysis. Nat. Methods *9*, 676–682.

76. Indra, A.K., Warot, X., Brocard, J., Bornert, J.M., Xiao, J.H., Chambon, P., and Metzger, D. (1999). Temporally-controlled site-specific mutagenesis in the basal layer of the epidermis: comparison of the recombinase activity of the tamoxifen-inducible Cre-ER(T) and Cre-ER(T2) recombinases. Nucleic Acids Res. *27*, 4324–4327.

77. Madisen, L., Zwingman, T.A., Sunkin, S.M., Oh, S.W., Zariwala, H.A., Gu, H., Ng, L.L., Palmiter, R.D., Hawrylycz, M.J., Jones, A.R., et al. (2010). A robust and high-throughput Cre reporting and characterization system for the whole mouse brain. Nat. Neurosci. *13*, 133–140.

78. Lin, B., Coleman, J.H., Peterson, J.N., Zunitch, M.J., Jang, W., Herrick, D. B., and Schwob, J.E. (2017). Injury Induces Endogenous Reprogramming and Dedifferentiation of Neuronal Progenitors to Multipotency. Cell Stem Cell *21*, 761–774.e5. https://doi.org/10.1016/j.stem.2017.09.008.

79. Ma, Q., Chen, Z., del Barco Barrantes, I., de la Pompa, J.L., and Anderson, D.J. (1998). neurogenin1 is essential for the determination of neuronal precursors for proximal cranial sensory ganglia. Neuron *20*, 469–482.

80. Wolf, F.A., Hamey, F.K., Plass, M., Solana, J., Dahlin, J.S., Göttgens, B., Rajewsky, N., Simon, L., and Theis, F.J. (2019). PAGA: graph abstraction reconciles clustering with trajectory inference through a topology preserving map of single cells. Genome Biol. *20*, 59.

81. Yates, B., Gray, K.A., Jones, T.E.M., and Bruford, E.A. (2021). Updates to HCOP: the HGNC comparison of orthology predictions tool. Brief. Bioinform. *22*, bbab155. https://doi.org/10.1093/bib/bbab155.

82. Bankhead, P., Loughrey, M.B., Fernández, J.A., Dombrowski, Y., McArt, D.G., Dunne, P.D., McQuaid, S., Gray, R.T., Murray, L.J., Coleman, H. G., et al. (2017). QuPath: Open source software for digital pathology image analysis. Sci. Rep. *7*, 16878.

STAR★METHODS

KEY RESOURCES TABLE

REAGENT or RESOURCE	SOURCE	IDENTIFIER
Antibodies		
Recombinant Anti-GAP43 antibody [EP890Y]	Abcam	ab75810; RRID:AB_1310252
Purified anti-Tubulin β 3 (TUBB3) Antibody (clone TUJ1)	Biolegend	801202; RRID:AB_2313773
UCHL1/PGP9.5 Polyclonal antibody	Proteintech	14730-1-AP; RRID:AB_2210497
SOX2 Monoclonal Antibody (Btjce)	Invitrogen	14-9811-82; RRID:AB_11219471
Human/Mouse NeuroD1 Antibody	R&D systems	AF2746; RRID:AB_2149217
Purified anti-Keratin 5 Polyclonal Chicken Antibody	Biolegend	905904; RRID:AB_2721743
Mouse anti-Mash-1	BD bioscience	556604; RRID:AB_396479
Rat anti-KI67	eBioscience	50245564; RRID:AB_10854564
Rabbit anti-Neurogranin	Sigma	AB5620; RRID:AB_91937
Rabbit anti-BSND	Abcam	ab196017; RRID:AB_2920894
Goat anti-OMP	Wako	544-10001-WAKO; RRID:AB_664696
Chemicals, peptides, and recombinant proteins		
murine R-spondin	Peprotech	#315-32
murine Noggin	Peprotech	#250-38
murine Wnt-3a	Peprotech	#315-32
Y27632	Tocris	#1254
Cultrex Stem Cell Qualified Reduced Growth Factor Basement Membrane Extract	Bio-techne	#3434-005-02
Critical commercial assays		
Evercode WT Mini v2 kit	Parse	ECW02010
Deposited data		
Single-cell RNAseq data	This study	GSE257536
Re-analyzed single-cell RNAseq data	Ualiyeva et al.[30]	GSE245074
Experimental models: Organisms/strains		
Wild-type adult C57/BL6 mice	Jax	stock #000664
Wild-type adult CD1 mice	Charles River	stock #022
K5-CreER^{T2}	P. Chambon via R. Reed, Jax	stock #018394
B6.Cg-Gt(ROSA)26Sortm9(CAG-TdTomato)Hez/J	Jax	stock #007909
Ascl1-TdTomato	Lin et al.[78]	RRID:MMRRC_043552-UCD
Neurog1-GFP BAC transgenic	Jax	stock #017306
Software and algorithms		
Scanpy	Wolf et al.[73]	https://github.com/scverse/scanpy
Scrublet	Wolock et al.[74]	https://github.com/swolock/scrublet
LIANA+	Dimitrov et al.[53]	https://github.com/saezlab/liana-py
FIJI/ImageJ	Schindelin et al.[75]	https://fiji.sc/

EXPERIMENTAL MODEL AND STUDY PARTICIPANT DETAILS

Wild-type adult C57/BL6 mice (WT) were purchased from Jax, stock #000664, and used for the organoid characterization experiments. Wild-type adult CD1 mice (CD1) were purchased from Charles River, stock #022, and used for the methodology

improvement experiments (aging and methimazole). Animals of both sexes were used in nearly all the experiments and were generally between 6 and 16 weeks of age. The only exception to this was in the methodology optimization experiments where all mice were male and between 3 and 6 weeks old. All mice were maintained following IACUC guidelines, with *ad libitum* rodent chow and water. The heat and humidity were controlled with a 12:12 h light-dark cycle. The Tufts IACUC approved all protocols using animals for the Humane Use of Animals at Tufts University School of Medicine, and all methods were performed in accordance with relevant guidelines and regulations. All animal work in this manuscript was done and reported following ARRIVE guidelines.

K5-CreER^{T2} (K5^{CreERT2}), transgenic mice that were generously provided by P. Chambon via R. Reed,[76] were bred to the Cre reporter (B6.Cg-Gt(ROSA)26Sortm9(CAG-TdTomato)Hez/J; stock #007909, which expresses TdTomato in HBCs after tamoxifen administration.[77] Ascl1-TdTomato mice were in-house,[78] MMRRC 043552, and expressed TdTomato in the Ascl1+ GBC stage. Neurog1-GFP BAC transgenic mice generated through the GENSAT Project were obtained from Jackson Labs, stock #017306.[79] Ascl1-TdTomato; Neurog1-eGFP dual reporter mice were obtained by breeding the Ascl1-TdTomato and Neurog1-eGFP mice together to simultaneously label the upstream Ascl1+ GBCs and the downstream Neurog1+ GBCs.

For the HBC lineage trace experiments, tamoxifen (diluted in corn oil, U.S.P. grade, 20 mg/ml, CAS 8001-30-7, Spectrum Ref. CO136, Lot. 2KA0296) was injected intraperitoneally at 150 mg/kg, and tissue was collected 5–7 days after tamoxifen injection. Methimazole (CAS: 60-56-0, diluted in PBS at 10 mg/mL) was injected intraperitoneally at 50 mg/kg, and tissue was collected between 1 and 14 days to generate a time course.

METHOD DETAILS

Tissue processing for cell culture

The animals were euthanized by CO_2 inhalation, followed by a transcardiac flush of 10–20 mL of 1x PBS (pH 7.4). The olfactory epithelium was harvested from the septum and both turbinates, avoiding the respiratory epithelium.[18] Tissue was minced in 500 μL of HBSS 1X (with calcium chloride and magnesium chloride, Gibco ref. 24020-117) before 500 μL of Papain (40U/ml in EBSS) and 500 μL of activation buffer (0.067 mM beta-mercaptoethanol, 1.1 mM EDTA, 5.5 mM Cystein-HCl, in EBSS) was added to the tissue and incubated for 30 min with agitation at 37°C. Samples were filtered through a 40 μm mesh, pelleted at 500g for 5 min), and washed twice with HBSS 1x or DMEM/F12 1x (with 1x primocin and Y27632 10μM, Gibco ref. 11320-033, lot. 2522615). The final pellet obtained was resuspended and cell-sorted or went straight to culture after counting. All steps are done on ice and performed as quickly as possible to avoid cell death.

Tissue processing for sections

The animals were euthanized by CO_2 inhalation, followed by a transcardiac flush of 10–20 mL of PBS 1x (pH 7.4) and 20 mL of fresh, ice-cold 4% paraformaldehyde. The nose was dissected, and the turbinates and septum were kept together. The tissue was kept in 4% PFA under vacuum for 1 h, followed by a wash with PBS 1x and left overnight in decalcification solution (Saturated EDTA, 4°C). The tissue was washed and left overnight in 30% sucrose and frozen in Scigen Tissue-Plus O.C.T. Compound (#23-730-571) using liquid nitrogen. Samples were stored at −80°C until they were cut into 12 μm coronal sections on a Leica cryostat.

Bulk RNA isolation

Animals were perfused with 10 mL of 1x PBS, and the nasal turbinates were dissected on ice. Tissue was then homogenized with 500 μL of Trizol followed by 100 μL of chloroform, which was vortexed for 30 s. Samples were then incubated for 3 min at room temperature (RT) and spun down for 15 min at 13,000g. The supernatant was collected, and 300 μL of RNAse-free isopropanol was added and incubated for 10 min at RT. The sample was then spun for 30 min at 4°C, 13,000 X g. The pellet was washed with RNAse-free 70% ethanol and resuspended in DEPC-treated water. cDNA was generated using Invitrogen SuperScript IV VILO Master Mix with ezDNase (#11766050) following the manufacturer's instructions.

Fluorescence-activated cell sorting (FACS)

Flow cytometry was performed on a FACS Aria II (BD Biosciences). Debris was excluded using forward, and side scatter area, and doublets were excluded using a stringent two-step gating based on forward and side scatter height versus width. Fluorescence-minus-one (FMO) controls guided gating schemes. Negative controls were used to gate away autofluorescence. The K5^{CreERT2} + HBC population and Ascl1-TdTomato GBC population were selected by the Tdtomato signal, and Neurog1-eGFP+ GBCs by the FITC signal. CD54/ICAM-1 sorting of HBCs was assessed and yielded non-viable HBCs, likely due to the blocking nature of the antibody on a surface adhesion receptor (data not shown).

Cell culture

Dissociated olfactory epithelial tissue was either FACS purified or plated into a tissue-culture-treated plate in DMEM/F12 media containing primocin for 1 h to remove fibroblasts. Afterward, the supernatant was collected, centrifuged, and diluted in DMEM/F12 media before counting and plating into chambers. Depending on the conditions used, different cell numbers were used per chamber

(2 wells: Lab-Tek #177380; 8 wells: Lab-Tek #177402; 4 wells: Lab-Tek #177399 or 8 wells: NEST #230108; 4 wells: NEST #230104), the specific cell number used for each experiment are described below.

For standard organoid culture and immunostaining, we used wild-type mice and plated 75,000 cells per well of an 8-well chamber slide. For single-cell RNAseq, 1.5 million cells were plated into 2-well chambers. For lineage trace experiments using K5^{CreERT2} lineage-traced mice, 75,000 cells were plated per well of an 8-well chamber slide. For the reconstitution experiments, 10,000 FACS-purified HBCs (K5^{CreERT2} mice, selected for TdTomato) and 10,000–67,000 Ascl1 and Neurog1 GBCs (eGFP+) were plated into each well. For the cell-contact transwell experiments, 75,000 cells of HBC-depleted cells were plated on the top chamber. 12,000 HBCs were plated on the bottom chamber or mixed in with the HBC-depleted cells. For the final chamber well experiment, either 75,000 of the HBC-depleted cells were plated, or 12,000 HBCs were additionally added to this population for the positive control.

For the reconstitution experiments, 1:1 and 1:5 mixing ratios of HBCs to Ascl1-GBCs were tested and yielded no statistically different results $p = 0.48$. For Neurog1-GBC reconstitution experiments, a 1:5 ratio was used.

Aging experiments used CD1 mice and were plated at a concentration of 75,000 cells/well of an 8-well chamber slide. The methimazole experiments also used CD1 mice, but loading per well was determined using a dilution curve between 25,000 and 100,000 cells/well, depending on the day after methimazole. For the methimazole HBC depletion experiment, 75,000 cells of each condition were plated. As a control, dissociated cells were run through the FACS protocol and machine.

Growth media

DMEM/F12 (Gibco #11320-33) was supplemented with recombinant murine R-spondin 1 (200 ng/mL, c #315-32); recombinant murine Noggin (100 ng/mL, Peprotech #250-38); recombinant murine Wnt-3a (50 ng/mL, Peprotech #315-32); Y27632 (10 μM, TOCRIS #1254); N2 (1%, Gibco #17502-048); B27 (2%, Gibco #17504-044); GlutaMAX 100x (1% vol/vol, Gibco #35050-061); HEPES 1M (10 mM, Gibco #15630-080); Cultrex Stem Cell Qualified Reduced Growth Factor Basement Membrane Extract (4% vol/vol with 9 mg/mL of protein, Bio-techne #3434-005-02).[26] Media was gently changed every other day, with care taken not to aspirate the Cultrex gel that forms on the bottom of the well.

Single-cell RNAseq

Organoids derived from naive wildtype CD1 mice were cultured in the standard organoid culture media described above. On day 7, 8 distinct chamber wells containing organoids were washed with ice-cold PBS and dissociated using TrypLE and Dispase until reduced to a single-cell suspension. Wells were pooled together. Single-cell RNAseq libraries were generated using the Evercode WT Mini v2 kit (Parse #ECW02010), following manufacturer instructions and loading 10,000 cells by manual counting. Libraries were sequenced using a Novaseq with a PE200 kit, cycles distributed as R1:146, I1:6, and R2:86. FASTQ files were processed using the Parse bioinformatics pipeline and mapped to the MM10 genome build and then analyzed using Scanpy.[73] Standard filtering was performed: only cells expressing at least 200 genes, greater than 800 unique UMI counts, and less than 2% mitochondrial reads were selected (removed 12.7% of cells). Counts were log-transformed. Doublets were removed using Scrublet at an automatic threshold of 0.44 doublet score, which removed 1.3% of the cells.[74] Clusters were first separated using leiden clustering at a resolution of 0.4, followed by one round of subclustering on the neurogenic cluster to separate GBCs from iOSNs. Clusters were manually identified using *a-priori* identified markers based on published literature. In all UMAP gene expression plots, gene expression is depicted as log-normalized counts. For plots focused on the neurogenic compartment, the entire cluster that had been identified using the leiden algorithm was subset and analyzed without recomputing UMAP coordinates. PAGA-based pseudotime[80] was computed using wrappers found natively in Scanpy. The Sox2+ cluster was designed as the root population based on published literature. For the transition plots based on pseudotime ordering, a log-normalized expression plot was provided along with a standardized-transformed expression plot to allow visualization of lowly-expressed genes. An olfactory receptor was determined to be "detected" when log10 normalized counts were >1.

FASTQ files and processed read counts are deposited at GEO accession: GSE257536.

For LIANA cell-cell signaling prediction, publicly available data was downloaded from GEO accession GSE245074.[30] The processed data already contained cell annotations, which were directly subset to include "HBC", "GBC", "Immature OSN", and "OSN." Aggregate LIANA+[52,53] was run using the mouse-converted cell-cell interaction database based on HCOP, the HGNC comparison of human to mouse gene conversions[81] with a cutoff of min_evidence = 3. For LIANA, a specificity cutoff of 0.01 was used.

Immunofluorescence

On D7 of culture, wells were washed with cold 1x PBS and fixed for 5 min with 4% PFA (made in 1x PBS, pH 7.2 to 7.3), washed with 1x PBS, followed by 15 min incubation with PBST (1x PBS, 0.05% Tween 20). Primary antibodies were incubated for 1h at RT or overnight at 4°C with gentle agitation, followed by 3 washes of PBST and incubation with secondary antibodies for an hour at RT. The following table shows the primary and secondary antibody dilutions in blocking buffer (1X PBS, 1% BSA, 0.3% Triton). The slides were mounted in fluoromont G and kept at 4°C or −20°C for more extended storage. Concentration of antibodies used and the cells they stain for are provided below.

Name	Abbreviation on the paper	Host	Brand/reference	Target	Dilution
Recombinant Anti-GAP43 antibody [EP890Y]	GAP43	Rabbit	Abcam/ab75810	Immature neurons	1:300
Purified anti-Tubulin β 3 (TUBB3) Antibody (clone TUJ1)	TUJ1	Mouse	Biolegend/801202	Immature neurons	1:3000
UCHL1/PGP9.5 Polyclonal antibody	PGP	Rabbit	Proteintech/14730-1-AP	Immature and mature neurons	1:800
SOX2 Monoclonal Antibody (Btjce)	SOX2	Rat	Invitrogen/14-9811-82	HBCs and multipotent GBCs	1:400
Human/Mouse NeuroD1 Antibody	NeuroD1	Goat	R&D systems/AF2746	GBC INP	1:200
Purified anti-Keratin 5 Polyclonal Chicken Antibody	K5	Chicken	Biolegend/905904	HBCs	1:2000
Mouse anti-Mash-1	Ascl-1	Mouse	BD bioscience/556604	Ascl-1 GBCs/gbc TA-OSN	1:100
Rat anti-KI67	KI67	Rat	eBioscience/50245564	Dividing cells	1:200
Rabbit anti-Neurogranin	NRGN	Rabbit	Sigma/AB5620	microvillar cells	1:200
Rabbit anti-BSND	BSND	Rabbit	Abcam/ab196017	ionocytes	1:300
Goat anti-OMP	OMP	Goat	Wako/544-10001-WAKO	mature OSNs	1:300
Various secondary antibodies		Donkey	Jackson Immuno		1:400 (reconstituted in glycerol)

QUANTIFICATION AND STATISTICAL ANALYSIS

Images were captured on a Zeiss 880 confocal microscope using optimal Z slice intervals. In all images, max-intensity projects of optical slices that contained the cells of interest are shown. For quantification, chamber slides were imaged using either a Keyence tiled imager or an Akoya Vectra3 automated imager. Image analysis was performed using FIJI/ImageJ (version 1.53).[75] Image preparation and figure assembly were performed in Adobe Illustrator (v 27.5). Only image brightness and contrast were altered in all photographs and applied to the entire image uniformly. For the % pixel by area, images were initially thresholded using the Otsu algorithm positive images before the calculated settings were reused for all data in a given experiment performed together on a single day. Thresholds were recalculated for each independent replicate. In cases where manual counting was necessary images were annotated and counted using QuPath.[82] In these cases, individual organoids were segmented based on the distinct physical separation of a well-integrated cluster of nuclei, where cell bodies were in direct contact with each other, while excluding axonal (Tuj1/PGP+) projections.

Numerical data was analyzed using GraphPad Prism 10.1.0. All data was tested for normality distribution using the Shapiro-Wilk test. All datasets passed the normality test and were analyzed using ANOVA, followed by Dunnett's post-hoc test. Statistical difference was considered when $p < 0.05$. The results are expressed as median (SE). For the methimazole qRT-PCR data, we used the delta-delta CT method for normalization and plotted the data as log2 fold-change. In certain experiments, we normalized the results, measured as a percentage Tuj1+ area, by the original cell input to control for differences in initial plating densities. These were then scaled by 100,000 to generate arbitrary units (a.u.). This was done for the mixing, contact, and methimazole experiments.

Cell Reports Methods, Volume *5*

Supplemental information

Quiescent horizontal basal stem cells

act as a niche for olfactory neurogenesis

in a mouse 3D organoid model

Juliana Gutschow Gameiro, Constantin A. Hintschich, Agnès Dekeyser, Valérie Hox, James E. Schwob, Eric H. Holbrook, Marco Aurélio Fornazieri, and Brian Lin

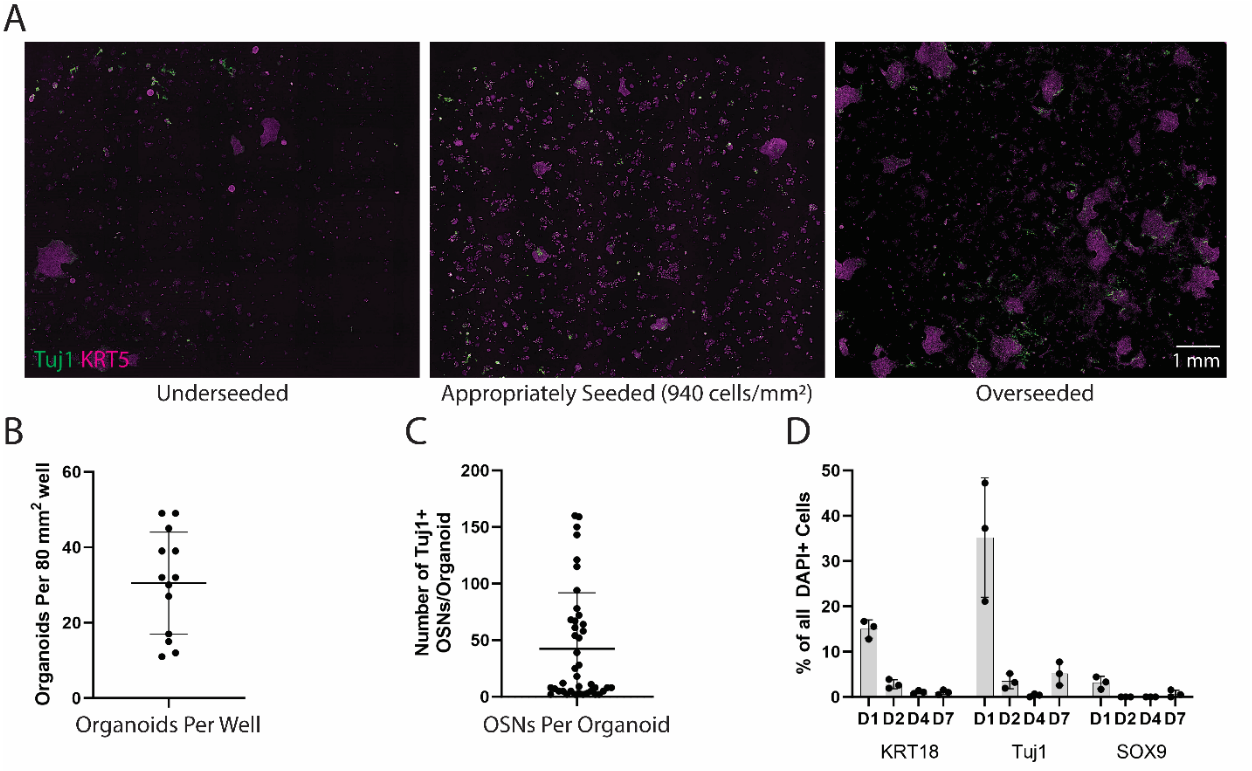

Figure S1: Optimization of organoid seeding and characteristics, related to Figure 1. (A) Representative images of organoid cultures that are underseeded, appropriately seeded, and overseeded. Images are of the entire well of an 8-well chamber slide. Underseeded conditions are uneven and have low numbers of organoids, while overseeded conditions have organoids that merge together, compromising the ability to quantify individual organoids. (B) Quantification of the number of organoids found per well of an 8-well chamber slide when seeded at 940 cells/ mm^2 across 4 distinct biological replicates. Each dot represents a single chamber well. (C) Quantification of the number of Tuj1+ nuclei found per organoid across four distinct biological replicates. Each dot represents a single organoid that was counted. (D) Quantification of cells that were KRT18+, Tuj1+, or SOX9+ in a timecourse starting the day after plating, shown as the percentage of all DAPI+ cells. Each dot represents a single chamber well, where random fields, which in total covered 25% of the well, were manually counted.

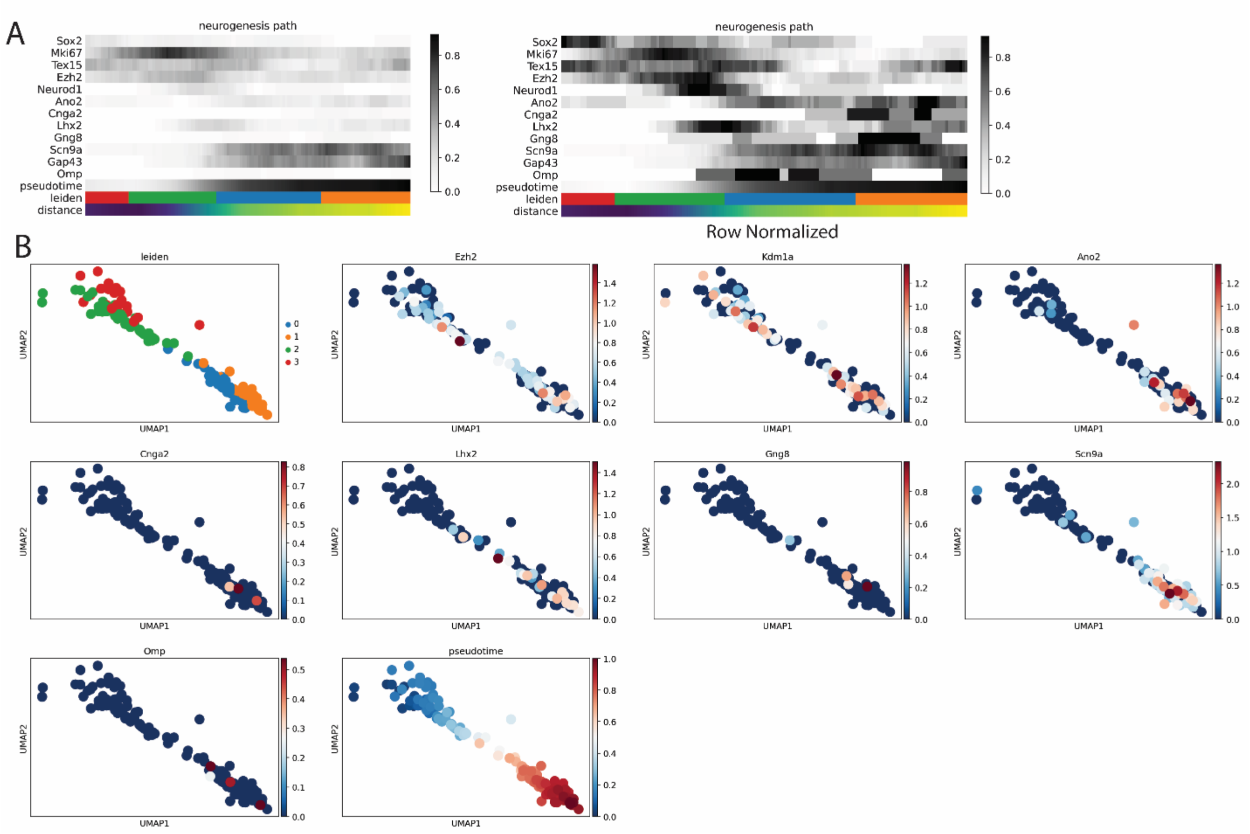

Figure S2: Single-cell RNAseq analysis, related to Figure 1. (A) Pseudotime-aligned gene expression plots of key neurogenic genes. Pseudotime was determined using PAGA, with the Sox2+ cluster set as the root population. On the left, log-transformed gene expression is plotted. On the right, data have been standardized to 0-1 expression to facilitate visualization of lowly expressed genes, which can also highlight noise, as can be seen in the OMP expression. (B) Gene expression of key genes found in neurogenesis and OSN function plotted on a log scale.

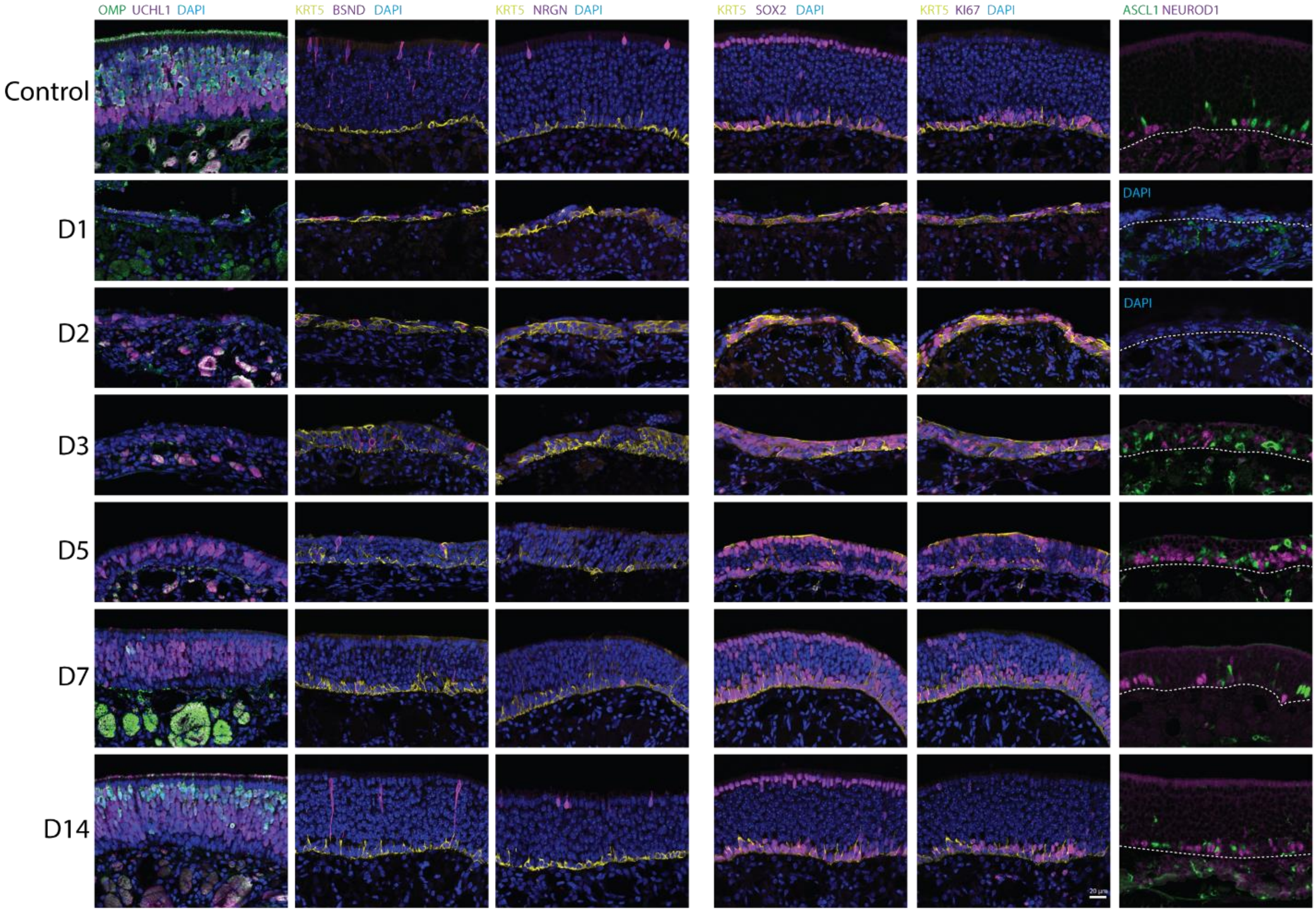

Figure S3: Characterization of methimazole-induced injury, related to Figure 1. Representative immunofluorescent staining for primary cell types found in the OE in a time course of tissue regeneration after methimazole injury. OMP marks mature OSNs, UCHL1/PGP9.5 marks mature and immature OSNs, KRT5 marks HBCs, BSND marks ionocytes/IP3R3+ microvillar cells, NRGN marks TRPM5+ microvillar/tuft cells, luminal SOX2 marks Sus cells, basal SOX2+ cells that are KRT5- are GBCs, KI67 marks dividing cells, ASCL1 marks neuronally committed upstream GBCs and NEUROD1 marks neuronally committed progenitor GBCs. In the right-most panels, DAPI is omitted except for D1 and D2 post-injury for clarity due to color overlap.

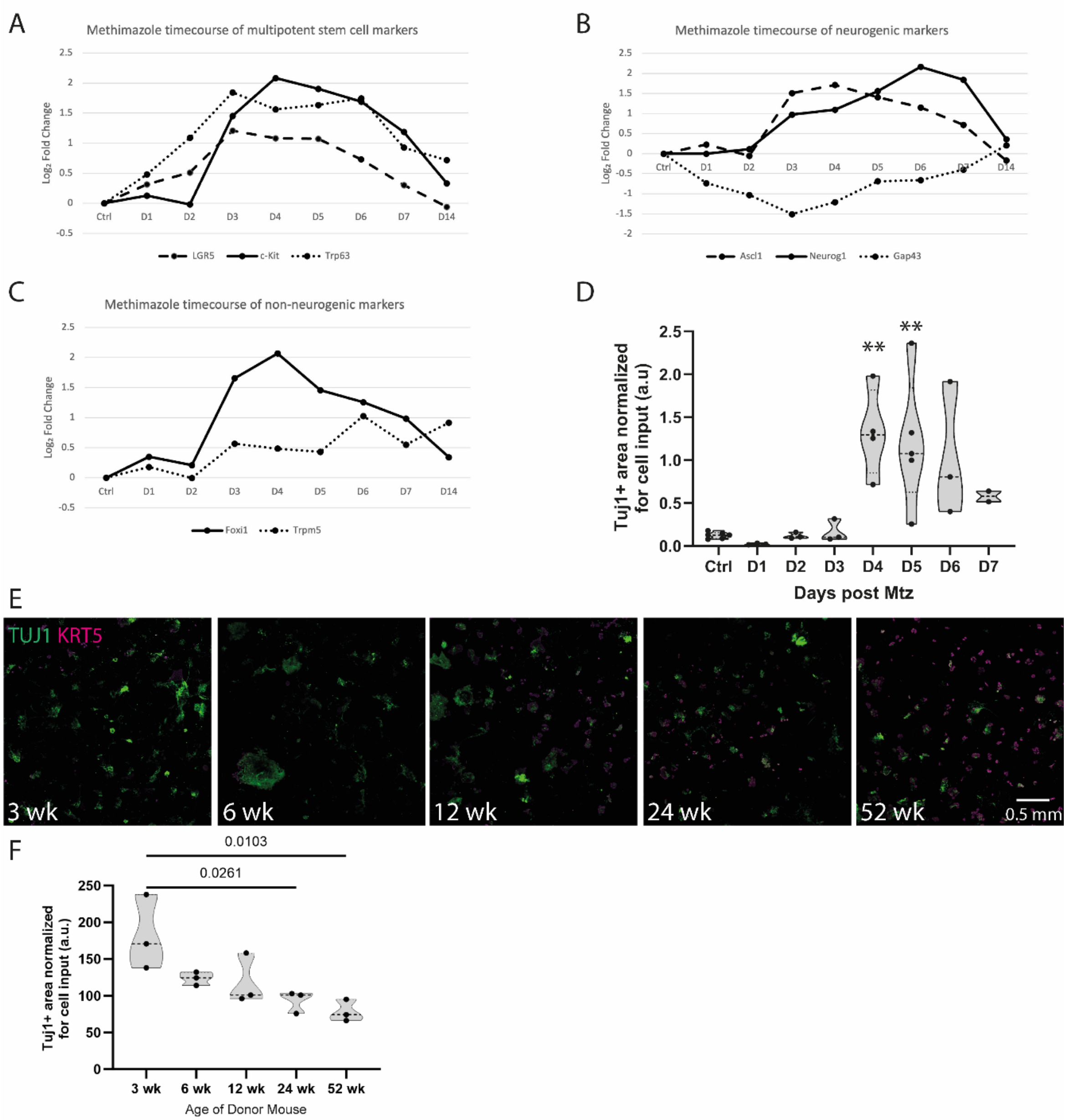

Figure S4: **Optimization of the organoid model, related to Figure 1.** q-RT PCR of OE tissue samples collected at time points post methimazole injury, profiling the gene expression of (A) multipotent stem cell markers, (B) neurogenic markers, and (C) non-neurogenic markers relative to control. (D) Quantification of the efficiency of neurogenesis in the organoid system using tissue harvested at different methimazole injury time points, measured as Tuj1+ area normalized for cell input (a.u.). ANOVA comparison $p = 0.001$, $^{**}p < 0.05$ compared to uninjured control). (E) Representative, low magnification images of organoid cultures stained for Tuj1 and KRT5 isolated from mice aged 3-52 weeks. (F) Quantification of the Tuj1+ area generated by cells isolated from mice of different ages, normalized for cell input (a.u.). ANOVA comparison $p = 0.013$, followed by Dunnett's multiple comparisons test. qRT-PCR n=1 mouse each timepoint, technical triplicates. Post-Mtz experiments were replicated a minimum of 3 times on distinct mice, with each dot representing a different mouse. Age experiments were replicated three times, with each dot representing a different mouse.

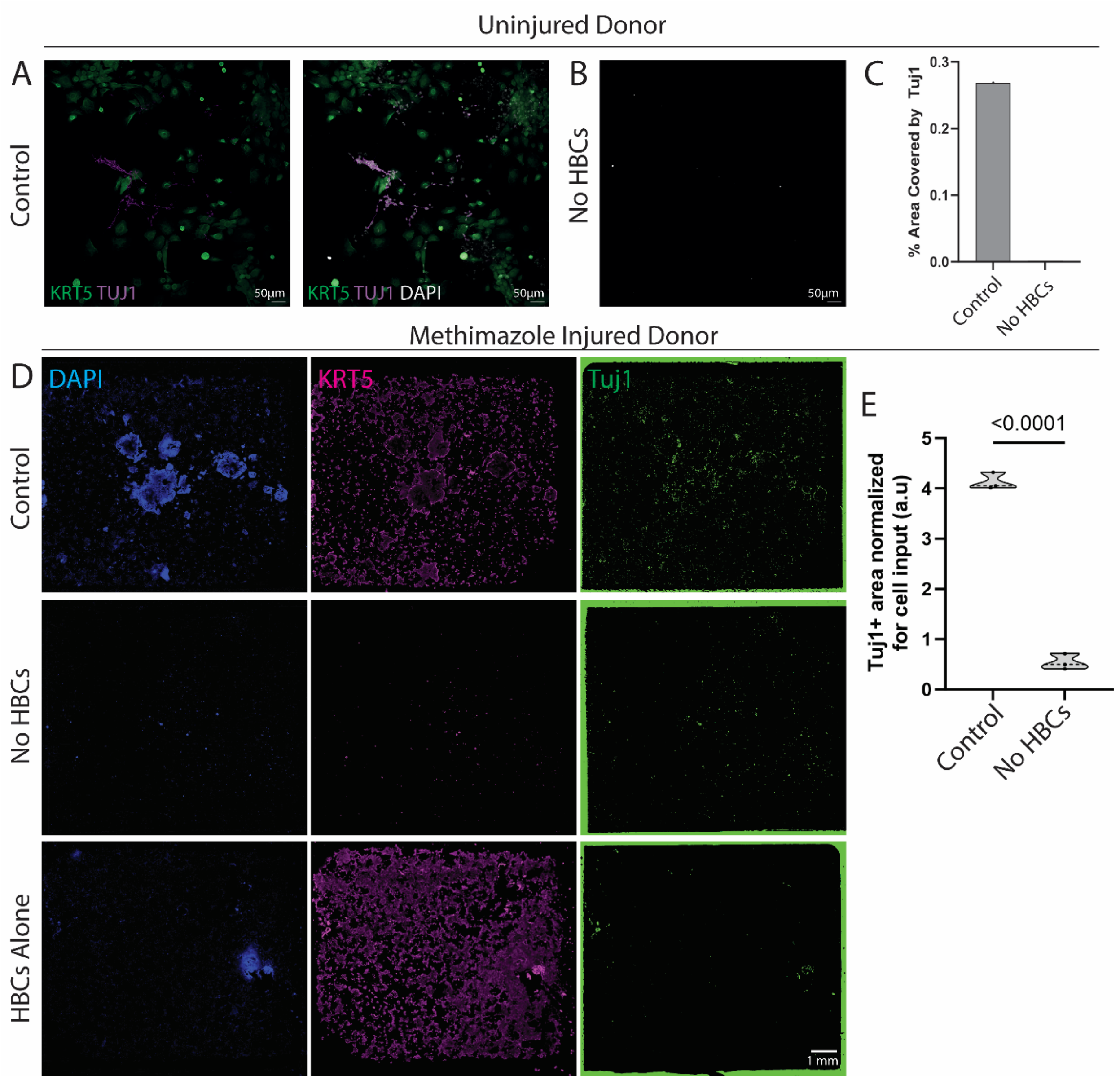

Figure S5: Effect of HBC depletion in chamber slides, related to Figure 3. (A) Representative immunostaining of the positive control (reconstituted HBCs and negative population that all went through the FACS process), and (B) immunostaining of HBC-depleted cultures grown in culture wells. (C) Quantification of the % area covered by Tuj1 in the chamber wells. (D) Representative whole chamber well images of cultures grown from donor animals 4 days after methimazole injury. Seeded cells were unstained controls that went through the entire FACS procedure, samples depleted for HBCs or sorted HBCs alone. Chambers were fixed and stained after 7 days of culture. The green border represents the auto fluorescent markings on the chamber slide itself. (E) Quantification of the Tuj1+ area normalized for chamber well area and cell input. Unpaired t-test, n=3 biological replicates.
